# High abundance of hydrocarbon-degrading *Alcanivorax* in plumes of hydrothermally active volcanoes in the South Pacific Ocean

**DOI:** 10.1038/s41396-023-01366-4

**Published:** 2023-01-31

**Authors:** Bledina Dede, Taylor Priest, Wolfgang Bach, Maren Walter, Rudolf Amann, Anke Meyerdierks

**Affiliations:** 1 Max Planck Institute for Marine Microbiology, Bremen, Germany; 2 MARUM, Center for Marine Environmental Sciences, University of Bremen, Bremen, Germany; 3 Geoscience Department, University of Bremen, Bremen, Germany; 4 Institute of Environmental Physics, University of Bremen, Bremen, Germany

**Keywords:** Microbial ecology, Molecular ecology

## Abstract

Species within the genus *Alcanivorax* are well known hydrocarbon-degraders that propagate quickly in oil spills and natural oil seepage. They are also inhabitants of the deep-sea and have been found in several hydrothermal plumes. However, an in-depth analysis of deep-sea *Alcanivorax* is currently lacking. In this study, we used multiple culture-independent techniques to analyze the microbial community composition of hydrothermal plumes in the Northern Tonga arc and Northeastern Lau Basin focusing on the autecology of *Alcanivorax*. The hydrothermal vents feeding the plumes are hosted in an arc volcano (Niua), a rear-arc caldera (Niuatahi) and the Northeast Lau Spreading Centre (Maka). Fluorescence in situ hybridization revealed that *Alcanivorax* dominated the community at two sites (1210–1565 mbsl), reaching up to 48% relative abundance (3.5 × 10^4^ cells/ml). Through 16S rRNA gene and metagenome analyses, we identified that this pattern was driven by two *Alcanivorax* species in the plumes of Niuatahi and Maka. Despite no indication for hydrocarbon presence in the plumes of these areas, a high expression of genes involved in hydrocarbon-degradation was observed. We hypothesize that the high abundance and gene expression of *Alcanivorax* is likely due to yet undiscovered hydrocarbon seepage from the seafloor, potentially resulting from recent volcanic activity in the area. Chain-length and complexity of hydrocarbons, and water depth could be driving niche partitioning in *Alcanivorax*.

## Introduction

At hydrothermal vents high-temperature fluids enriched in reduced chemical compounds rise from the seafloor and mix with ambient seawater until reaching a depth of neutral buoyancy, where the plume spreads laterally [1]. Vents exert a global impact by influencing the food web dynamics and biogeochemical cycles beyond the area of venting [2–4]. Depending on the geological setting, the chemical composition of plume varies, e.g., sulfur-rich plumes are characteristic of basalt-hosted systems, while hydrogen- and methane-rich plumes are characteristic of serpentine-hosted systems [1]. Indicatory of the plumes’ chemistry are the structures and functions of microbial communities [5].

As the research on microbial communities of hydrothermal systems has become more extensive, the *Alcanivorax* genus has been shown to inhabit several plumes [6–9]. Enrichment of *Alcanivorax* has been reported in deep waters of the Mariana Trench (up to 17.8%; ref. [10]), where serpentine-hosted systems are observed, as well as in the plume water from basalt-hosted systems of Mid-Ocean Ridge [6]. Although the *Alcanivorax* genus has been found across the oceans, it predominantly inhabits oil-contaminated waters, where they exhibit a hydrocarbon-degrading lifestyle [11]. The type strain *Alcanivorax borkumensis* SK2 was reported to have an impaired metabolic response to hydrostatic pressure [12], however, other *Alcanivorax* species were determined to account for up to 10% of the microbial community in the bathypelagic layer [10]. Experiments on pure cultures [13–15] have revealed a capability of *Alcanivorax* to utilize glutamic acid, pyruvic acid, sucrose, mannose and others [16] and to accumulate storage lipids [17]. Li et al. [10] proposed that their presence in the deep sea is linked to oil seepage and the proximity to alkane-releasing serpentinite mud volcanoes in the forearc of the Mariana subduction zone, however, these data were inconclusive. An in-depth analysis of the *Alcanivorax* genus in plumes of deep-sea spreading-ridges and non-ridge volcanoes is currently lacking.

In this study, we analyzed the microbial communities inhabiting hydrothermal plumes of neovolcanic features of Niua, Niuatahi and Maka in the Tonga arc, rear-arc and the North-Eastern Lau Spreading Centre, which is one of the most tectonically and magmatically active zones on the planet [18]. The microbial communities were investigated by using a polyphasic approach, including 16S rRNA gene amplicon analysis, metagenomics, metatranscriptomics and fluorescence in situ hybridization (FISH). We report two distinct community structures in Niuatahi and Maka plumes. The communities of the rising, buoyant plumes were rich in chemosynthetic SUP05 bacteria, whilst *Alcanivorax* dominated the laterally spreading, non-buoyant plumes. Using the retrieved metagenome-assembled genomes (MAGs), we are able to pinpoint a depth-derived *Alcanivorax* niche in the deep-sea and build a hypothesis regarding their high abundance.

## Methods

### Site description

Four hydrothermal vents fields, Niua South, Niua North, Niuatahi, and Maka in the Southwest Pacific (Fig. S1) were sampled during R/V Sonne expedition SO263 in July 2018. Niua is a volcanic edifice in the northernmost part of the Tonga arc. Niua South vents 325 °C hot, metal-rich fluids poor in sulfide, methane and H_2_ [19–21]. The Niua North vent fluids are characterized as white smoker-type fluids, since they are acidic and have moderately high sulfide contents [19, 22]. Niuatahi (previously known as Volcano O) is a very large rear-arc caldera volcano (~15 km diameter), mid-way between the North Eastern Lau Spreading Centre (NELSC) and the Tonga arc. The Niuatahi caldera is characterized by dacitic lavas and hosts several active hydrothermal systems, including North caldera, and sites in the south and southwest of the caldera as well as acid-sulfate venting from a central volcanic cone [19, 23, 24]. Maka volcano, discovered in 2008 by Nautilus Minerals Inc., is located at the NELSC [23]. Maka’s vent fluids are sulfide-rich (>30 mM) [19, 22, 25], whereas Niuatahi has moderate to low concentrations of sulfide (~4 mM), CH_4_ (>0.4 µM) and H_2_ (~0.8 µM) [19].

Plume samples were collected with a SPE-32 carousel water sampler attached to a conductivity, temperature and depth (CTD) device. Samples were collected based on detected changes in turbidity and redox potential during four vertical CTD casts in Niua (NiuaS-Site1), Niuatahi (Nn-Site5, Ns-Site6) and Maka (M-site8), and four tow-yo CTD casts across the Niua (NiuaS-Site2, NiuaN-Site3, NiuaN-Site4) and Niuatahi caldera (Ns-Site7) (Table S1, Fig. S1). Early rising plumes (*n* = 4) were sampled a few meters from the orifice using Niskin bottles mounted on the remotely operated vehicle (ROV) Quest (MARUM, Bremen). Early rising plume samples were collected from the Niuatahi North caldera, 6 m above focused hot fluid emission (sample Nn-1ROV-rp), and the south-central zone, 2.2 m above the orifice (sample Ns-2ROV-rp). At Maka, the ROV sampled plume water close to a black smoker (T_max_: 329 °C) (sample M-4ROV-rp) and above a mussel field (sample M-3ROV-rp). An additional CTD cast was run close to Mata volcano, 20 km from Niua North [17]. The CTD data did not indicate the presence of a plume at the sampled depths. Therefore, the samples serve as background samples in this study (B-Site1; B-Site2) (Fig. S2). In addition, primordial ^3^He is used to define the samples taken in relation to plumes (Table S2) and to extrapolate helium concentration in the end-member fluid. He isotope ratios (R/R_A_) are reported as the He isotope ratio of the sample (R = ^3^He/^4^He) in relation to the atmospheric ratio (R_A_ = (^3^He/^4^He)_atmospheric_ = 1.39 × 10^−6^). Sample collection and measurements of helium data is given in detail in the Supplementary Information and Tables.

Samples were processed within 1 h of the CTD being retrieved. Details on sampling procedure are given in Supplementary Information and Tables, and Fig. S3. For early rising plume samples, 700–900 ml of water were filtered through 0.22 µm pore-size polycarbonate (PC) membrane filters (47 mm diameter, Millipore, Darmstadt, Germany) and preserved at −80 °C for downstream meta-omics analyses. Large volume samples (10–20 L) collected during CTD-rosette casts were filtered through 142 mm diameter PC filters (0.22 µm pore size). Filters were subsequently sliced and preserved at −80 °C for further analysis. An additional 500 ml of water collected during the CTD casts was fixed with formaldehyde (1% final concentration) for 10–14 h at 4 °C for FISH analysis. Afterwards, cells were collected by filtration on 0.22 µm pore size PC membrane filters, dried and frozen at −20 °C until use.

### DNA extraction and 16S rRNA gene analysis

DNA was extracted either from one half of a 47 mm diameter filter (ROV sample), or from a 2 cm^2^ piece of a 142 mm diameter filter (CTD sample) using the PowerSoil DNA Isolation Kit (MoBio, Ca, USA), according to the manufacturer’s instructions. DNA concentration was determined using a Qubit 3.0 Fluorometer (Invitrogen, Darmstadt, Germany). The V3–V4 region of the 16S rRNA gene was amplified using primer combination Bakt_341F and Bakt_805R [26] as described elsewhere [9]. Equimolar amounts of PCR product were pooled. Amplicons were sequenced in paired-end mode (2 × 250 bp) on a HiSeq 2500 sequencer (Illumina, San Diego, CA, USA) at the Max-Planck-Genome-Centre (Cologne, Germany).

### Meta-omics sequencing

The extracted DNA of sample Nn-Site5-wp and Ns-Site6-bp was used for metagenome analysis. The protocol “Ovation Ultralow System v2 1-16” (NuGEN, CA, USA) was followed for end-repair, ligation and purification. The fragments were sequenced in paired-end mode (2 × 250 bp length) on a HiSeq 2500 sequencer (Illumina) at the Max-Planck-Genome-Centre (Table S3).

Total RNA was extracted from 10 cm² membrane filter pieces (Nn-Site5-wp and Ns-Site6-bp) according to a previously published protocol [27]. Technical replicates of both samples were extracted and sequenced. An Illumina-compatible library was generated with the NEBNext Ultra II Directional RNA Library Prep kit (NEB, Ipswich, MA, USA) followed by sequencing on a HiSeq 3000 system (Illumina) in paired-end mode (2 × 150 bp length) with 3.67 × 10^7^–4.66 × 10^7^ reads being generated per sample.

### ASV analysis

Raw read fastq files were demultiplexed using cutadapt v1.15 [28] and processed in R (R version v4.0.3; ref. [29]) following the DADA2 v1.16.0 pipeline [30] (https://benjjneb.github.io/dada2/tutorial.html), with the modification: filterAndTrim, truncLen=c(227,227), pool=TRUE. Taxonomic and species-level assignments were performed using the DADA2-formatted SILVA SSU Ref NR99 138 database. Alpha diversity was calculated using phyloseq v1.32.0 [31]. Non-metric multidimensional scaling (NMDS) analysis was performed using the ordinate function with a Bray–Curtis dissimilarity matrix as an input in R [29]. Principal component analysis (PCA) was also conducted in R. Endmember fluid concentrations of H_2_, CH_4_ and H_2_S were taken from the MARHYS database v1 [32] for a comparison between fluid samples.

16S rRNA gene sequences assigned to the *Alcanivorax* genus were aligned to a curated version of the SILVA 138 database [33] (alignment quality >85, pintail >50) using SINA [34]. Phylogenetic trees were constructed based on 30 cultivated representative *Alcanivorax* 16S rRNA gene sequences (quality >98) in ARB [35]. Several treeing algorithms, neighbor-joining [35], PhyML [36] and RAxML [37] and different position conservation filters (no filter, 20%, 30%, 40%) were applied. The DADA2-generated *Alcanivorax* ASVs were added to the trees using maximum parsimony, without allowing changes of the overall tree topology.

### CARD-FISH

Catalyzed reporter deposition-FISH (CARD-FISH) analysis was performed as previously described [38]. Probes used in this study: EUBI-III [39, 40], NON338 [41], ARCH915 [42], GAM42a with competitor BET42a [43], *Alteromonas* - ALT1413 [44], a combination of *Alcanivorax* probes: ALV735, ALV735-b and ALV461 [45], SAR202-312R [46], and SUP05_1241 [9]. A mix of ALV735 and ALV735-b probe was used for enumeration of all *Alcanivorax*, since the probes did not sufficiently discriminate between the different subgroups. ALV461, with a more specific target range, was used for visualization of species affiliated with the *A. borkumensis* branch of the 16S rRNA gene tree. Two helper probes were designed for ALV735 (CCGCCTTCGCCACTGGTGTTCCT; GCTCCCCACGCTTTCGCACCTCAG) and two helper probes for ALV461 (ATAAGCCTTCCTCCCTACT; CCCACGCTTTCGCACCTCAG). Probes targeting the *Alcanivorax* genus and their helpers were used with 10% formamide concentration for specific binding at 46 °C. Filters were counted using a Image 2D (Zeiss, Jena, Germany) or Eclipse 50i (Nikon, Tokyo, Japan) microscope. Imaging was done with a laser scanning microscope (LSM780) equipped with an Airyscan detector (Zeiss).

### Metagenome analysis

#### Sequence analysis pipeline

Raw reads were quality trimmed using BBDuk of the BBtools package v35.14 [47] with a quality score threshold of 20 and a minimum length of 100 bp. The quality of the reads was assessed using FastQC v0.11.7 [48]. Nonpareil v3.303 [49] was used to estimate the coverage level of the metagenomes. All metagenomic raw reads were taxonomically assigned using Kaiju v1.6.2 [50]. 16S rRNA genes were sorted using SortMeRNA v2.0 [51] and taxonomically classified using SILVAngs (SILVA SSU Ref dataset, release 138) [33]. Metagenomes were assembled with MEGAHIT v1.2.9 [52] using k-mer steps of 10 and a maximum k-mer size of 127. Assembly quality was assessed using QUAST v5.0.2 [53] before being individually binned using CONCOCT v1.1.0 [54] in Anvi’o v6.1 [55]. Moreover, targeted reassembly, as described in Meier et al. [9], was performed on *Gammaproteobacteria* MAGs using the SPAdes assembler v3.10.1 [56]. After two to three reassembly rounds, several MAGs with a completeness >50% were retrieved. In addition, a coassembly was performed with the metagenomes of this study and one previously published from Brothers volcano plume, sample 49CTD_b16 [27]. This resulted in the generation of two additional MAGs (Alc-5 and Alc-6). Completeness and contamination of the MAGs was estimated by CheckM v1.1.2 [57]. All MAGs were visualized and manually refined using the anvi-interactive interface of Anvi’o.

### MAG analysis

A total of 15 medium and high quality MAGs were retrieved (Table S4), according to MIMAG standards [58]. GTDB-Tk was used to assign taxonomic classification to MAGs [59]. MAGs affiliated with *Alcanivorax* were functionally characterized. Initial gene prediction for each MAG, was performed by Prokka v1.14.6 [60]. Carbohydrate-active enzymes and TonB-dependent receptors were predicted as described previously [61].

A genome-based maximum likelihood phylogenetic tree of *Alcanivorax* MAGs was calculated using an alignment of 120 bacterial marker genes by HMMER [62] in GTDB-Tk v1.0.2 [59] (Release89). High- and intermediate-quality *Alcanivorax* MAGs from the Genome Repository of Oiled Systems (GROS) [63] were added to the phylogenetic tree of *Alcanivorax* genomes. The tree was visualized using iTOL [64]. Average amino acid identity (AAI) of retrieved MAGs was calculated by CompareM v10.1.1 (https://github.com/dparks1134/CompareM). Viral genes were mined using PHASTER [65]. Analysis of the core and pan-genomes of *Alcanivorax* MAGs and closely related cultivated species were performed, following the pangenomics workflow in Anvi’o.

### Metatranscriptome sequencing and analysis

Metatranscriptomic paired-end reads were quality trimmed using Trimmomatic v0.39 [66] with a minimum length threshold of 100 bp and quality threshold of 15, using a 4 base sliding window. 16S rRNA reads were sorted using SortMeRNA [51] and taxonomically classified using SILVAngs (SILVA SSU Ref dataset, release 138) [33]. Remaining mRNA reads were subsequently mapped to the MAGs using BBMap of the BBtools package (parameters: minid=97) [47] and the values normalized into transcripts per million (TPM) using Geneious Prime 2019.2.3.

In order to determine total MAG abundance and activity, metagenomic and metatranscriptomic raw reads were mapped to MAGs using BBMap (minid=99). Reads per kilobase per million (RPKM) was calculated based on MAG length and number of reads mapped. The activity of each MAG was determined as the average TPM of four house-keeping genes present in all analyzed MAGs: DNA gyrase subunit A, RecA protein, DNA-directed RNA polymerase beta subunit and leucyl/phenylalanyl tRNA protein transferase [67].

### Biogeography of *Alcanivorax*

In order to assess the distribution of *Alcanivorax*, reads from the globally-distributed surface and mesopelagic metagenomes of the Tara Oceans dataset (PRJEB1787) [68] and bathypelagic metagenomes of the Malaspina expedition [69] were recruited to the MAGs using BBMap (minid=99%). Raw reads of metagenomes from plumes of Lau Basin (Tui Malila, Tahi Moana, Kilo Moana, Mariner and Abe) [70], Kermadec arc (Macauley and Brothers) [27] and Mid-Atlantic Ridge (Woody Crack vent at Menez Gwen) [9], and other hydrocarbon-rich environments (Table S5) were also recruited to *Alcanivorax* MAGs. As described in Karthikeyan et al. [63], TAD80 (Truncated Average sequencing Depth) was calculated after mapping a subset of Tara Oceans and Malaspina metagenomes to *Alcanivorax* MAGs (20 metagenomes of Tara Ocean and 20 metagenomes of Malaspina). By comparing TAD80 values with RPKM, we defined thresholds to determine the presence of MAGs, thus removing false positives. These thresholds were 0.04 RPKM in Tara Oceans metagenomes and 0.2 RPKM in Malaspina Expedition metagenomes (Fig. S4).

### Data accession

Raw reads and the Metagenome Assembled Genomes were submitted to the European Nucleotide Archive under project number PRJEB49968. The 16S rRNA dataset is submitted under project number PRJNA821857.

## Results

### Amplicon sequence variants

Twenty plume samples were collected at four sites in the Tonga arc (Niua North—three samples, Niua South—four samples, Niuatahi—ten samples, Maka—three samples), each with different plume properties and intensities. They were compared to four early rising plume samples from Niuatahi (Nn-1ROV-rp and Ns-2ROV-rp) and Maka (M-3ROV-rp; M-4ROV-rp) and two background (B) samples (Table S1; Fig. S1). Sample names consist of: (1) the name of the vent (Niua South – NiuaS; Niua North – NiuaN; Niuatahi north caldera – Nn; Niuatahi south caldera – Ns; Maka – M), (2) the type which refers to CTD cast (Site) or ROV dive and (3) sample category: such as: p – non-buoyant plume, ap – above non-buoyant plume, bp – below non-buoyant plume; wp – weak non-buoyant plume and rp – rising plume (Table S1). Turbidity (Table S1) and Helium excess (δ ^3^He) in the plume water (Table S2) were used as hydrothermal tracers in order to assign sample categories. The extrapolated R/R_A_ of end-member vent fluids was in the range of 7.0–7.5 at Niua North and South. The obtained R/R_A_ of Niuatahi’s end-member vent fluid was 7.8, while the R/R_A_ of the Maka vent fluid was 9.0.

**Fig. 1:**
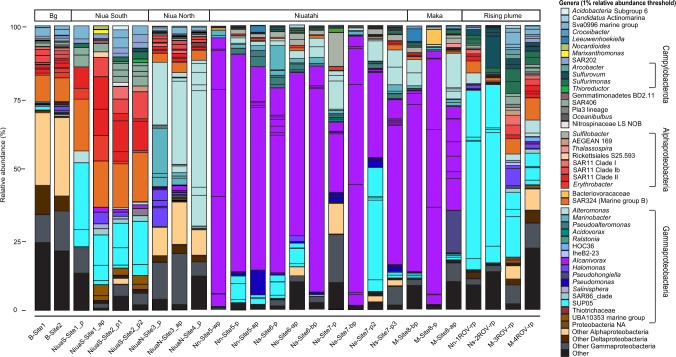
Relative abundance of 16S rRNA gene amplicon sequence variants (ASVs). The barchart depicts the taxonomic assignment of all ASVs annotated at the level of genera with 1% relative abundance threshold. Samples are grouped based on the properties of environment sampled, such as: background, plumes of different hydothermal sites such as: Niua North and South, Niuatahi and Maka and rising plume of Niuatahi and Maka. The ASVs were analyzed via a DADA2 pipeline [30].

An initial assessment of the microbial diversity was performed using 16S rRNA gene amplicon sequence variants (ASVs) (Fig. 1). The datasets for early rising plume communities were dominated by reads assigned to Gammaproteobacteria (23–68%) and Campylobacterota (8–19%) (formerly Epsilonproteobacteria; ref. [71, 72]). At higher taxonomic resolution, most of the reads were assigned to the SUP05-clade (Gammaproteobacteria; up to 63%), and the genera *Sulfurovum* (Campylobacterota; up to 15%) and *Sulfurimonas* (Campylobacterota; up to 9%).

In all later plume stages, Gammaproteobacteria dominated the microbial communities, fluctuating from 45 to 95% of reads. However, differences were observed at higher taxonomic resolution across different vent fields. Niua North datasets were dominated by reads affiliated with *Alteromonas* (≤43%) and *Marinobacter* (≤19%), whereas the Niua South plume was characterized by a community rich in SUP05 (≤36%), SAR11 (≤27%) and SAR324 (≤18%). In contrast, at Niuatahi and Maka, most of the reads were assigned to only two genera: *Alcanivorax* (Niuatahi: 24–94%; Maka: 37–89% of reads) and SUP05 (Niuatahi: up to 42%; Maka: ≤2% of reads). In addition, in Niuatahi and Maka, other prominent gammaproteobacterial ASVs were assigned to *Alteromonas* (up to 16%), *Marinobacter* (up to 10%) and *Pseudoalteromonas* (up to 6%).

Altogether, in Niuatahi and Maka, 45 ASVs were assigned to the genus *Alcanivorax*. Of these, only six reached more than 1% relative read abundance at both sites. A 16S rRNA gene-based phylogenetic tree confirmed the affiliation of these with the *A. venustensis* and *A. borkumensis* branch (Fig. S5). ASVs affiliated with the ‘*A. venustensis*-branch’ of the tree constituted the majority of reads in Nn-Site5-wp (86%), Ns-Site7-bp (86%), M-Site8-p (83%) and M-Site8-ap (19%) (Fig. S6). In contrast, two ASVs phylogenetically affiliated with the ‘*A. borkumensis*-branch’ (Fig. S5) dominated the rest of the plume samples, reaching up to 76% in Nn-Site5-p and 60% in M-Site8-bp (Fig. S6). The *A. venustensis*-related ASVs 3, 4 and 5 shared high similarity (99.5%), and occurred at a similar ratio in every sample, likely reflecting different rRNA operons within the same species. The background samples were dominated by reads assigned to Alphaproteobacteria (≤26%), mainly to SAR11 (≤10%), SAR324 (≤9%), other Gammaproteobacteria (≤12%) and Sva0996 (≤4%) (Fig. 1).

Non-metric multidimensional scaling (NMDS) analysis showed a clear differentiation between Niua South and North samples. Niuatahi and Maka plume samples were clearly separated from Niua and early rising plume as well as background samples (Fig. S7). In addition, PCA of 232 endmember fluids’ concentration of CH_4_, H_2_S and H_2_, taken from the MARHYS database [32], revealed a clear separation between Niuatahi and Maka and other volcanic, spreading zone and back-arc basin fluids (Fig. S8).

### Microscopic analyses

Total cell counts ranged from 2.12 × 10^4^ to 3.75 × 10^4^ in Niua, 4.63 × 10^4^ to 6.69 × 10^4^ in Niuatahi and from 2.23 × 10^4^ to 2.85 × 10^4^ in Maka plume (Fig. S9).

A dominance of bacterial (70% - Ns-Site6-bp) over archaeal cells (45% – Ns-Site7-bp) was determined using CARD-FISH. In addition, the relative abundance of *Alteromonas*, SUP05 and *Alcanivorax* was determined, as they were the most abundant clades detected by ASV analysis. The highest abundance of *Alteromonas* was determined in Niutahi south cone (Ns-Site7-bp - 2%). The *Alteromonas* ASVs fell in an uncultured clade within Alteromonadaceae, which is possibly not targeted by the probe. The SUP05_1241 probe detected the highest abundance of SUP05 in NiuaS-Site1-p (43%), supporting 16S rRNA gene analysis (Fig. S9). The abundance of *Alcanivorax* species was difficult to determine since they formed aggregates in Nn-Site5-wp, but were detected as single cells in Ns-Site6-bp. Single *Alcanivorax* cells exhibited a relative abundance of 25% (M-Site8-p) to 48% (Ns-Site6-bp). Using the ALV461 probe, the aggregates could be assigned to species on the ‘*A. borkumensis* branch’ of the phylogenetic tree (Fig. S5).

### Meta-omics analyses

The high abundance of *Alcanivorax* in hydrothermal plumes at two of the four investigated sites (Niuatahi and Maka) as well as the clear dominance of one or the other species at the *Alcanivorax*-rich sites was unexpected. Therefore, we investigated the presence and expression of functional traits in the weak plume sampled at Niuatahi north (Nn-Site5-wp; *A. venustensis*-rich) and below plume at Niuatahi south (Ns-Site6-bp; *A. borkumensis*-rich) by analyzing metagenomes and metatranscriptomes.

In agreement with the amplicon analysis, 16S rRNA genes extracted from the metagenomes were affiliated mainly with *Alcanivorax* in Nn-Site5-wp (74%) and Ns-Site6-bp (50%). A high expression of *Alcanivorax*-affiliated 16S rRNA genes was also identified in the metatranscriptomes, wherein they represented 21.0% and 53.5% of 16S rRNA genes identified in Nn-Site5-wp and Ns-Site6-bp (Fig. S10).

#### Metagenome assembled genomes

**Fig. 2:**
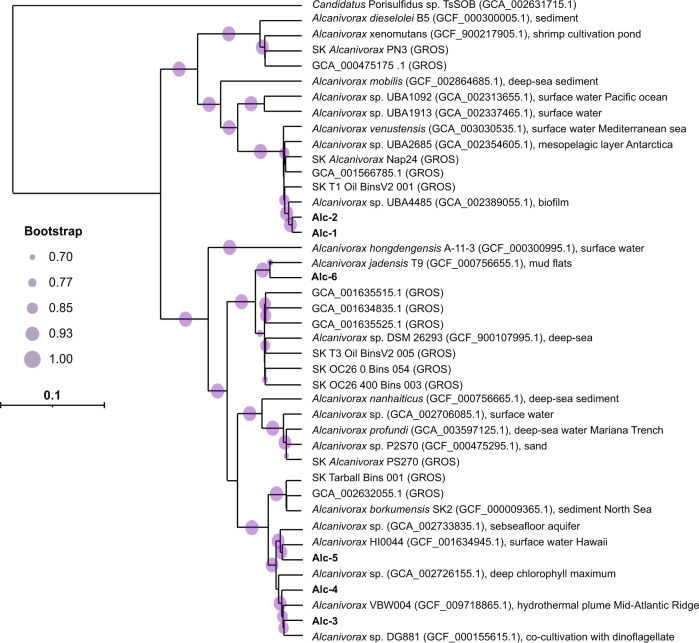
Phylogenetic analysis of the *Alcanivorax* genomes. The phylogenetic tree is based on an alignment of 120 bacterial marker genes from *Alcanivorax* MAGs included in GTDB [59], fourteen MAGs from GROS [63] and six MAGs retrieved in this study. The tree was calculated using GTDB-Tk [59]. The isolation source of the cultivated species is given next to the accession numbers. *Ca*. Porisulfidus was used as an outgroup. The two subgroups exhibited a clear difference in GC content, <60% for *Alcanivorax venustensis*-related genomes (upper branch) and >63% for *A. borkumensis*-related genomes (lower branch).

Altogether, six *Alcanivorax*-affiliated MAGs were retrieved (Alc-1 to Alc-6), with a completeness >89% and contamination <5.7% (Table S4). Among those, Alc-1 and Alc-2 shared >98% average AAI and >96.7% with the cultivated species *Alcanivorax venustensis*. Alc-3 and Alc-4 shared 98% AAI between each other and >97.5% AAI with the cultivated species *Alcanivorax* DG881 (GCA_000155615) and *Alcanivorax* VBW004 (GCA_009718865) (Fig. S11) [73, 74]. *Alcanivorax* DG881 and *Alcanivorax* VBW004 are closely related to *Alcanivorax borkumensis*. Alc-5 and Alc-6 shared >98% AAI with the cultivated species *Alcanivorax* HI0044 and *Alcanivorax jadensis*, respectively (Fig. S11). Phylogenetic tree reconstruction based on MAGs and genomes of the GTDB and GROS database, revealed a clear separation into two subclades within the *Alcanivorax* genus (Fig. 2). These two subclades exhibited a clear difference in GC content, <60% for *Alcanivorax venustensis*-related genomes and >63% for the *A. borkumensis*-related genomes (data not shown). The subclade separation was supported by 16S rRNA gene-based phylogenetic tree reconstruction including MAG derived 16S rRNA gene sequences (Fig. S5).

**Fig. 3:**
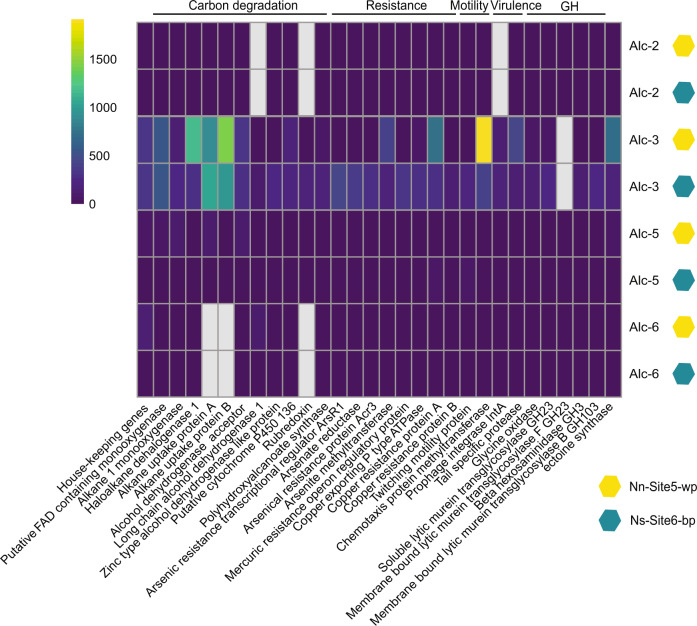
Expression level of genes in *Alcanivorax* MAGs. Reads were recruited from each metatranscriptome, Nn-Site5-wp and Ns-Site6-bp, to the MAGs using BBmap with a 97% minimum identity threshold. Transcripts were normalized by gene length and the total number of reads in each metatranscriptome (TPM). Values shown represent the average TPM derived from two metatranscriptome technical replicates. Gray cells indicate that the gene is missing in the MAG. GH is an abbreviation for glycosyl hydrolases.

The *Alcanivorax venustensis*-related Alc-2 and the *Alcanivorax* DG881-related Alc-3 were the most complete and least contaminated MAGs of each clade and therefore, used for further analysis. Alc-2 was abundant in the Nn-Site5-wp metagenome (141.9 RPKM; Fig. S12) compared to Ns-Site6-bp (0.5 RPKM), but exhibited in both samples a low expression of house-keeping genes (0.05 TPM in Nn-Site5-wp; 1.53 TPM in Ns-Site6-bp) (Fig. 3). In contrast, Alc-3 dominated the metagenome of Ns-Site6-bp (112.4 RPKM) compared to Nn-Site5-wp (7 RPKM), but exhibited significant expression of house-keeping genes in both samples (Ns-Site6-bp: 162 TPM; Nn-Site5-wp: 126 TPM). Alc-5 and Alc-6 were present at low abundance in both samples (<2 RPKM) and exhibited low expression (0.045 TPM in Nn-Site5-wp; <2 TPM in Ns-Site6-bp).

##### Genomic functional repertoire of Alcanivorax species

In order to assess conserved and unique functional genes, a pangenomic analysis was performed for *Alcanivorax* species using Alc-1 to -6, and closely related published MAGs and reference genomes from GTDB (Fig. S13). This comparative analysis showed that 1378 genes constituted the core *Alcanivorax* genome. An additional 137 genes were only shared between Alc-1, Alc-2 and the closely related genome of *Alcanivorax venustensis*, but were missing from all other MAGs. Among the genes harbored exclusively by these three genomes were genes for choline transporter and the flagellum system. These three genomes also harbored a higher number of transporters (Fig. S14). In contrast, Alc-3, Alc-4, *Alcanivorax* DG881 and *Alcanivorax* VBW004 had no annotated group characteristics. Across all genomes the highest number of peptidases was detected in Alc-6 and the closely-related *Alcanivorax jadensis* (Fig. S14).

##### Metabolic capabilities of Alcanivorax MAGs

To determine if differences in the genomic repertoire of the *Alcanivorax* MAGs (Alc-1 to -6) could explain the observed dynamics, the presence of metabolic pathways and resistance genes was investigated. All retrieved MAGs encoded type IV pili, twitching type II pili and either part, or all (Alc-2 and Alc-1) genes for chemotaxis (data not shown). A whole repertoire of heavy-metal resistance genes was present in every MAG, including arsenic, cadmium, cobalt, zinc and copper resistance genes (Fig. S15). Alc-1 and Alc-2 contained mercury reductase genes flanked by two transposases (Fig. S16). Genes coding for enzymes of chemotrophic pathways including sulfur, hydrogen, carbon monoxide, manganese and methane oxidation were not detected in these MAGs. Genes for key enzymes in carbon fixation via Calvin-Benson-Bassham and reverse TCA cycle as well as the reductive acetyl-CoA pathway were also not found. All *Alcanivorax* MAGs contained genes encoding proteins of the assimilatory sulfate reduction and the assimilatory nitrate reduction pathway. These MAGs harbored genes for glutamate, glucose and glycerol degradation, while the genes for degradation of starch, ascorbate, mannose and sucrose were missing. In addition, all MAGs contained genes for alkane monooxygenase, alcohol dehydrogenase and cytochrome P450, involved in short and mid length alkane degradation (C5-C17). Putative flavin-dependent monoxygenases, responsible for the degradation of long alkanes (C32+), were encoded by all MAGs (Fig. 3). In Alc-3, a putative FAD-containing monooxygenase was located between two transposases (Fig. S17). A gene related to the intracellular storage of carbon, polyhydroxyalkanoate synthase (*pha*Cd), was present in all MAGs. All MAGs lacked the acetyltransferase (*atfA1*) involved in the synthesis of storage lipids, including triacylglycerols (TAGs) and wax esters.

We further annotated several transporters of metals, vitamins, amino acids, sulfur, nitrogen, phosphorus and others (Fig. S18). Alc-3 contained ten consecutive alkane uptake transporters. Noteworthy was the presence of a nitrate and nitrite transporter only in Alc-6, but not in the closely-related cultivated species *Alcanivorax jadensis*. All MAGs encoded genes for transport and synthesis of osmoprotectants, including glycine betaine transporters as well as ectoine synthases and transporter (Fig. 3). Moreover, all MAGs contained genes for biosurfactant biosynthesis which participate in glycerolipid, glycerophospholipid, proline metabolism, bacteria secretion system and lipoprotein transport [75]. In addition, 10 transposases and two prophages with a 24 kb length (36 genes) and a 9.3 kb length (10 genes) were integrated in Alc-3 (Fig. S19).

##### Expressed functional repertoire of Alcanivorax MAGs

The most expressed functional genes within Alc-3 were related to heavy-metal resistance and alkane uptake and degradation (Fig. 3). However, differences were observed between the two samples. In Nn-Site5-wp, among the most expressed genes were the ones involved in haloalkane degradation and resistance: e.g., haloalkane dehydrogenase (1164 TPM), copper resistance (752 TPM) and arsenite methyltransferase (393 TPM). In contrast, in Ns-Site6-bp, alkane uptake (1052 TPM), twitching motility genes, intracellular carbon storage genes (up to 150 TPM), biosurfactant production genes (up to 250 TPM; Fig. S20), as well as putative FAD-containing monooxygenase (540 TPM) involved in the degradation of C32 + hydrocarbons were more expressed than in Nn-Site5-wp.

A clear distinction in gene expression patterns was also identified for Alc-5 in the two samples. In sample Ns-Site6-bp, heavy-metal defence genes were among the most expressed (up to 45 TPM), whereas in Nn-Site5-wp, the alkane degradation pathway was highly expressed (190 TPM). Alc-2 and Alc-6, both, exhibited low gene expression in the two samples (Fig. 3).

### Biogeography of *Alcanivorax*

**Fig. 4:**
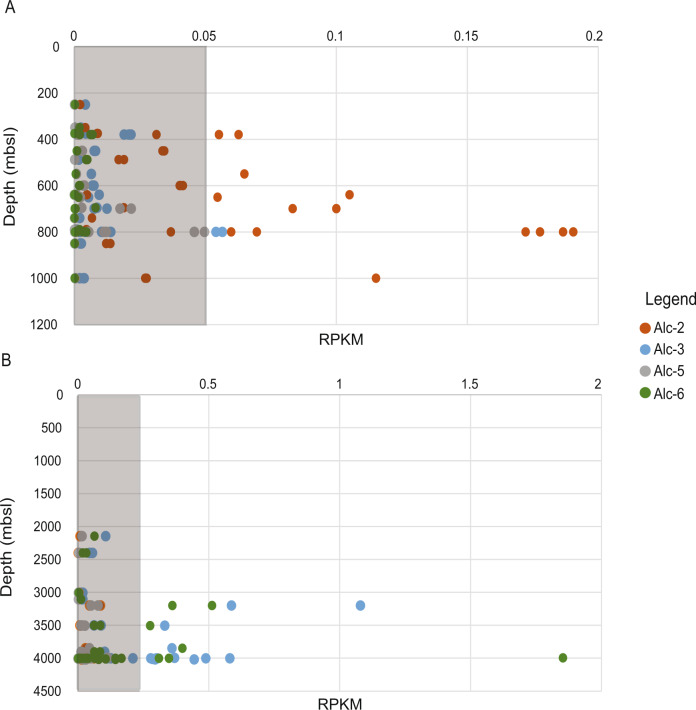
Mapping of TARA Oceans and Malaspina metagenome datasets to *Alcanivorax*-MAGs. **A** Mapping of Tara Oceans metagenomes. **B** Mapping of Malaspina metagenomes. The gray bar represents the threshold at which MAG presence in the metagenome was not reliable according to TAD80 [63].

Presence of MAGs affiliated with *Alcanivorax* was assessed in other hydrothermal plumes, oil spills and the surface and mesopelagic TARA Oceans metagenomes and bathypelagic Malaspina Expedition metagenomes (Table S5). The highest RPKM values of Alc-2 were identified between 400 and 1000 mbsl (up to 0.19 RPKM), whereas for Alc-3 and Alc-6, the highest values were observed in the bathypelagic layer below 2000 mbsl (up to 1.8 RPKM) (Fig. 4, Fig. S21). Alc-2 and Alc-3 were also abundant in Mariana Trench (0.23 RPKM). Alc-5 had the highest abundance in plumes of Brothers volcano (Brothers_49CTD_b16: 5.8 RPKM) and Mariner (0.15 RPKM). Altogether, these *Alcanivorax* MAGs were not present in oil spill environments and hydrocarbon-rich plumes and sediments (Fig. S22).

## Discussion

The genus *Alcanivorax* has caught the attention of scientists in recent years, not only due to their capabilities for bioremediation but as an indicator of hydrocarbon pollution [76]. In addition to accidental spills, *Alcanivorax* has been reported to inhabit the plumes of many seeps and hydrothermal vents, of different depths and geochemical characteristics [6, 9, 10, 70, 74]. In this study, the microbial communities of four deep-sea hydrothermal vent sites of the yet sparsely studied Tonga Arc were investigated for the first time. Intriguingly, multiple cultivation-independent techniques indicated a dominance of *Alcanivorax* at two of the sites, and the high expression of genes involved in hydrocarbon degradation, despite having no indication of hydrocarbons being contained in hydrothermal fluids released from Niuatahi and Maka volcanoes.

### *Alcanivorax* in Tonga Arc plumes

In contrast to ultramafic-hosted hydrothermal vents (e.g., Mid-Atlantic Ridge) or organic-rich vents (e.g., in the Guaymas Basin), which are known for their high hydrocarbon concentration in fluids and plumes, the geological setting of the Tonga Arc does not suggest significant hydrocarbon release from the vents. Consistent with this notion, the methane concentrations measured in the vent fluids were rather low (<10 µM; ref. [19]). Accordingly, the microbial communities at both sites at Niua are indicative of the systems. The samples of the white smoker plume of Niua North, collected above a sulfur pit covered with microbial mats, are dominated by non-hydrocarbonoclastic heterotrophic bacteria (*Alteromonas* and *Marinobacter*; ref. [9]). Although white smokers are observed across many ocean basins, the microbial communities inhabiting their plume are understudied. As they are characterized by a slower flow rate [77], the community could be expected to resemble the diluted diffusive flow, rich in heterotrophic bacteria [78]. Niua South plume samples, on the other hand, obtained from above the black smokers, reflect a typical community of a sulfide-rich plume with high abundance of SUP05 [27].

The microbial communities in Niuatahi and Maka plumes, however, were dominated by members of the *Alcanivorax*, well known for their important role in hydrocarbon degradation [11]. Such a community structure appears relatively unique to Niuatahi and Maka (Fig. S22). A cross-comparison between the concentration of CH_4_, H_2_S and H_2_ in 232 endmember fluids revealed that the Niuatahi and Maka fluids differ from those venting from other arcs, backarcs, and mid-ocean ridges, since they have high H_2_S and rather low H_2_ und CH_4_ content (Fig. S8). The high H_2_S in the plumes would usually support a SUP05-rich population comparable to those observed at Niua South or Brothers volcano at the Kermadec Arc [27], however, this was not the case.

The proportion of *Alcanivorax* in microbial communities in these two plumes is intriguing, since it is much higher than that identified in oil spills. This is potentially due to how the oil spills were sampled, i.e., in a late stage of the spills, which propagates other types of hydrocarbonoclastic bacteria, capable of degrading different hydrocarbons [11, 79, 80].

**Fig. 5:**
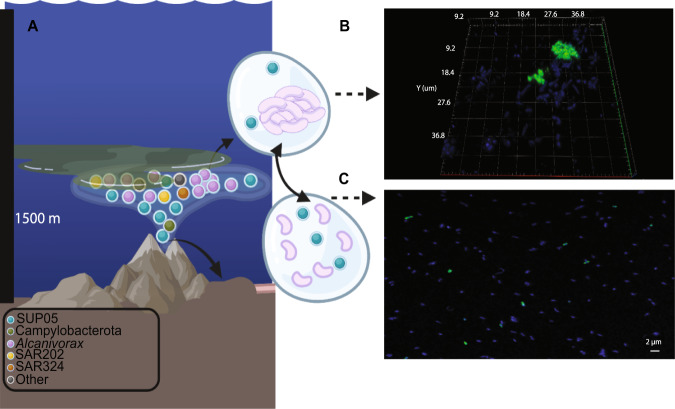
Features of the Niuatahi plume’s microbial community. **A** Depiction of the microbial community composition in the rising plume and plume samples. Circles represent the most abundant taxa clarified on the legend on the left hand site. **B** *Alcanivorax* aggregates visualized in 3D by ALV461 probe in Nn-Site5-wp sample. **C** *Alcanivorax* single cells visualized by Alc461 probe in Ns-Site6-bp sample. Green - CARD-FISH signal; blue - DAPI signal. Imaging was done with a laser scanning microscope (LSM780) equipped with an Airyscan detector. Figure created with BioRender.com.

In this study, *Alcanivorax* 16S rRNA read frequency accounted for up to 90% of the bacterial community. This may likely be an overestimate (Fig. S23), due to the presence of multiple 16S rRNA operons described for *Alcanivorax* spp. [81]. Furthermore, accurate cell quantification by CARD-FISH was compromised by aggregate formation in some samples (e.g., at Niuatahi North/Nn-Site5-wp; Fig. 5). Nevertheless, conservative estimates for Ns-Site6-bp indicated 3.5 × 10^4^ cells/ml (48% of the community), an abundance which has not yet been reported for any other deep-sea sample [10]. We here report four independent lines of evidence—16S rRNA gene analysis, CARD-FISH, metagenomics and metatranscriptomics—which all indicate a high presence and activity of *Alcanivorax*, accounting for about one half of the bacterial community.

### Global distribution of *Alcanivorax* MAGs

*Alcanivorax* spp. were present at very low abundance in the early rising plume of Niuatahi and Maka (up to 0.5%), but were abundant in all other plume samples from these sites, with no correlation to the available environmental parameters (data not shown). *Alcanivorax* MAGs did not contain genes related to chemotrophic processes, such as sulfur, hydrogen, manganese or methane oxidation. Therefore, the detected species seemed not to be related to plume age or intensity.

The *Alcanivorax* spp. within the plume communities of Niuatahi and Maka, consisted of four different species (*Alcanivorax* *venustensis* - Alc-2, *Alcanivorax* DG881 - Alc-3, *Alcanivorax* HI0044 - Alc-5 and *Alcanivorax jadensis* - Alc-6). The global distribution of Alc-2 (*Alcanivorax venustensis*) and Alc-3 (*Alcanivorax* DG881) in the mesopelagic TARA oceans metagenomes and the Malaspina dataset (Fig. 4), indicated a clear depth-derived niche: Alc-2 was up to threefold more abundant than other retrieved *Alcanivorax* MAGs in the mesopelagic layer (200–1000 m), whereas Alc-3 and Alc-6 were tenfold more abundant in the bathypelagic layer (2000–4000 m). Comparison of functional traits between the most abundant Alc-2 and Alc-3 elucidated some key differences. One difference is indeed associated to hydrostatic pressure. To maintain intracellular turgor pressure, bacteria adapt to abiotic changes, such as salinity, either through accumulation or biosynthesis of small organic compounds [12, 82, 83]. Both MAGs harbor genes for the biosynthesis of ectoine, which could be used as a piezolyte against hydrostatic pressure [12]. However, in Alc-2, potentially due to genome rearrangement, gene synteny is lost, as the ectoine pathway genes are found across different contigs, and gene expression is low. Alc-3 on the other hand, contains an operon-like organization of genes for the ectoine biosynthesis pathway, which is highly expressed in Nn-Site5-wp.

In addition, Alc-3 harbors ten consecutive alkane uptake transporter genes, more than *Alcanivorax borkumensis* (5–6 constitutive alkane uptake transporter genes; ref. [84]). Both MAGs, Alc-2 and Alc-3 contain genes for the degradation of C5-C17 hydrocarbon chains, however, only Alc-3 has a high expression of a Flavin-binding monooxygenase, which is located between transposases (Fig. S17). Flavin-binding monooxygenases are reported to degrade C32 + hydrocarbon molecules [85], meaning that Alc-3 abundance could have been impacted by the length and complexity of hydrocarbons in the plume. This is also supported by the high expression of carbon intracellular storage genes and genes encoding biosurfactant production in the sample Ns-Site6-bp in Alc-3 (Fig. S20). In addition, the complexity of hydrocarbons in the fluid could have driven the formation of the cell aggregates in Nn-Site5-wp. The non-surface attached bacterial aggregates affiliated to Alc-3 could be responsible for the low yield of DNA extraction and consequently the underestimation of the Alc-3 in Nn-Site5-wp. Based on the distribution and expression patterns in our samples, which were retrieved from ~1500 mbsl, we hypothesize that in Niuatahi and Maka plumes, we are witnessing a selection of *Alcanivorax* species driven by depth and the complexity of hydrocarbons.

### Implications for carbon cycling

The presence and the possible origin of hydrocarbons in these plumes remains unclear. Our fluid sampling detected only low concentrations of CH_4_ [19], and hence the hydrocarbons were most likely not originating from the focused hot venting we observed. This is supported by the fact that *Alcanivorax* abundance was also high in samples with weak plume signal (e.g., those sampled below or above neutrally-buoyant plume peaks). Additionally, there have been no reports of an oil spill and there are no major shipping routes that could have introduced hydrocarbons in this area. However, there is abundant sediment accumulations in the northeastern Lau Basin which could potentially lead to hydrocarbon release upon sill intrusions similar to the Guaymas Basin. Although quite speculative, seismic survey results suggest that the so called oil kitchens may exist in Eocene rocks south and west of Tongatopu, Tonga [86]. Oil companies had previously explored the presence of petroleum in the region, however, the attempts were unsuccessful [86]. Nonetheless, the high magmatic activity of the area [87, 88], could have created a hydrocarbon seepage in the proximity of Niuatahi and Maka, which is fueling the *Alcanivorax* community at selected sites. Potential recent volcanic activity is corroborated by the high ^3^He/^4^He ratios detected in the plume waters. The extrapolated high R/R_A_ of the end member fluids of Maka and Niuatahi indicate an increased magmatic activity at the time of sampling. Even taking into account the uncertainties involved in extrapolating endmember values, the observed R/R_A_ are substantially higher than the previously calculated values at Maka (2009) and Niuatahi (2012) [89]. We assume that the previously reported R/R_A_ values are lower than depleted mantle, i.e., Mid-Ocean Ridges [90, 91], due to radiogenic ingrowth of ^4^He in the crust. The increased R/R_A_, which we detected, could hence mean influx of mantle melts feeding crustal magma reservoir that provide the heat source for hydrothermal circulation.

The high expression of haloalkane dehalogenase genes, supports the presence of haloalkanes, volatile chemicals that could be produced by volcanic eruptions [92]. Thus, the *Alcanivorax* presence could be linked to volcanic activity causing the release of hydrocarbons. Our study sites are far from the subduction zone and there is no indication of serpentinite mud volcanoes in the Tonga forearc. Hence, we rule out a subduction-related influx of hydrocarbons, as proposed in Li et al. [10], for the Tonga arc.

The observation and chemical characterization of volcanic eruptions and their related plume events are rare, due to the short-lived nature of these plumes and the limited resources to sample the remote areas [93, 94]. To understand the real extent of the hydrocarbon presence in this region and the wider deep-sea, further exploration and research needs to be conducted. Based on the distribution and expression of *Alcanivorax* species reported in this study, other locations such as the south of the Liliput hydrothermal vent (26°54'36.0“S; 21°25'48.0“W), the Southern Indian ocean (29°48'36.0“S; 82°37'12.0“E) and the Great Australian Bright (39°13'48.0“S; 135°11'24.0“E), strike as areas of interest regarding hydrocarbon leakage (Table S6). For future ocean sampling campaigns, elevated abundances of *Alcanivorax* species could act as an indicator to pinpoint areas where hydrocarbon-seepage could be occurring.

## Conclusion

By using a multidisciplinary approach, we provide evidence that hydrocarbon-degrading *Alcanivorax* dominated the plumes of two volcanoes in the Tonga arc. The high abundance of two *Alcanivorax* species, *Alcanivorax* DG881 and *Alcanivorax venustensis*, coincided with the high expression of hydrocarbon-degrading genes, despite having no indication of hydrocarbon presence in the hydrothermal fluid. Therefore, we hypothesize that *Alcanivorax* population could be fueled by hydrocarbon leakage happening in the proximity of these vent systems, potentially due to volcanic activity. The length and complexity of hydrocarbons could be driving a niche partitioning between the two species. Another important factor playing a role in *Alcanivorax* DG881 and *Alcanivorax venustensis*’ global distribution could be water depth. This study presents evidence that microbes could be used as indicators of environmental perturbation and particularly the high abundance of *Alcanivorax* could point us toward hydrocarbon leakages.

## Supplementary information

Supplementary Information and Tables

Supplementary Figures
