# Supplementary material for: High abundance of hydrocarbon-degrading Alcanivorax in plumes of hydrothermally active volcanoes in the South Pacific Ocean
Source: ISME J. 2023 Jan 31;17(4):600–10. doi: 10.1038/s41396-023-01366-4 (PMC10030979; doi:10.1038/s41396-023-01366-4)
Supplement: Supplementary file 1 — Supplementary Information and Tables [file 41396_2023_1366_MOESM1_ESM.pdf]

Supplementary Information and Tables

Sample collection

Firstly, after retrieval of the CTD onboard, subsamples for FISH analyses were collected directly from the outlet of the Niskin bottle into 500 ml glass bottles (Fig. S3). The water in the 500 ml bottle was immediately fixed using formaldehyde to preserve community composition. After 12 hours in the fridge, the water was filtered onto PC filters, which were dried and stored for FISH analysis.

Secondly, Niskin bottles were emptied into 10 L plastic (PE) containers. The 10 L containers were transported to the lab where the water was filtered using a peristaltic pump. While one sample was filtering, the next sample was emptied from the Niskin bottle to another 10 L container and was further processed. The processing time was kept within 1 hour at maximum from CTD-rosette retrieval on board to filter storage in the -80°C freezers. The filtration device, containers, bottles and water hoses were cleaned 3 times with ~1 L MilliQ prior to each usage.

Helium data

Samples for noble gas analyses were taken directly from Niskin bottles attached either to the CTD or the ROV in pinched-off copper tubes, while avoiding contamination by air bubbles. High-resolution sector field mass spectrometer (MAP 215-50, Mass Analyser Products, Manchester UK) was used to measure the ratio of ^3He to ^4He. In addition, a quadrupole mass spectrometer (Balzers QMG 112a) was used for analyses of Ne and He concentrations (1).

The excess of primordial ^3He of a sample is derived from the $^3He/^4He$ ratio (R) of that sample in relation to the atmospheric ratio of $^3He/^4He$ (R_A=1.39x10^{-6}), and is reported as δ^3He, which is $(R/R_A-1)*100$. Since no samples for noble gas analysis could be taken from the end-member fluid, the helium isotopic composition of the vent fluids were derived by extrapolating the $^3He/^4He$

ratio to a Ne/He ratio of zero (2). Ne in the plume waters is predominately atmospherically derived, therefore the undiluted end-member fluid has negligible Ne concentrations, i.e. zero-Ne/He.

Data accession

Raw reads and the Metagenome Assembled Genomes were submitted to the European Nucleotide Archive (ENA) under project number PRJEB49968. The 16S rRNA dataset is submitted under project number PRJNA821857. Raw data of physical oceanography during SO253 cruise is published in PANGAEA https://doi.org/10.1594/PANGAEA.949874 (3).

Table S1: Samples analyzed in this study. Given are the sampling location, determined physical and chemical parameters (4), and the experiments conducted on the different samples during this study (MG: Metagenomics, MT: Metatranscriptomics, FISH: fluorescence *in situ* hybridization). Nd- not determined.

Sample	Real sample name	Location	Charac-teristics	Latitude	Longitude	Depth [m]	Temp [°C]	O_2 [ml/l]	Turbi-dity (△NTU)	MG/MT/ FISH
B-Site1	01CTD_b14	Background	No signal	-15.06	-173.65	700	5.7	2.75	0.24	-/-/-
B-Site2	01CTD_b10	Background	No signal	-15.06	-173.65	1102	3.8	2.7	0.25	-/-/-
NiuaS-Site1-p	06CTD_b9	Niua South	Plume	-15.164	-173.574	1022	4.1	2.7	0.29	-/-/+
NiuaS-Site1-ap	06CTD_b13	Niua South	Above plume	-15.164	-173.574	953	4.6	2.66	0.25	-/-/-
NiuaS-Site2-p1	12CTD_b1	Niua South	Plume	-15.163	-173.574	1041	4.0	2.64	0.26	-/-/+
NiuaS-Site2-p2	12CTD_b7	Niua South	Plume	-15.168	-173.574	1100	3.9	2.7	0.26	-/-/+
NiuaN-Site3-p	35CTD_b2	Niua North	Plume	-15.081	-173.558	699	5.6	2.9	0.24	-/-/+
NiuaN-Site3-ap	35CTD_b12	Niua North	Above plume	-15.081	-173.553	639	5.9	3.2	0.24	-/-/+
NiuaN-Site4_p	36CTD_b2	Niua North	Plume	-15.072	-173.547	740	5.6	2.9	0.24	-/-/+
Nn-Site5-wp	87CTD_b3	Niuatahi north	Weak plume	-15.327	-173.996	1554	2.75	2.8	0.26	+/+/+
Nn-Site5-p	87CTD_b7	Niuatahi north	Plume	-15.327	-173.996	1502	2.9	2.77	0.26	-/-/+
Nn-Site5-ap	87CTD_b13	Niuatahi north	Above plume	-15.327	-173.996	1402	3.0	2.8	0.25	-/-/-
Ns-Site6-p	88CTD_b6	Niuatahi south	Plume	-15.388	-173.997	1462	2.9	2.8	0.34	-/-/-
Ns-Site6-ap	88CTD_b8	Niuatahi south	Above plume	-15.388	-173.997	1402	2.9	2.8	0.27	-/-/-
Ns-Site6-bp	88CTD_b9	Niuatahi south	Below plume	-15.387	-173.997	1252	3.2	2.8	0.25	+/+/+
Ns-Site7-p	73CTD_b2	Niuatahi south	Plume	-15.399	-174.005	1210	3.4	2.85	0.25	-/-/-
Ns-Site7-bp	73CTD_b5	Niuatahi south	Below plume	-15.397	-174.004	1300	3.13	2.8	0.25	-/-/+
Ns-Site7-p2	73CTD_b7	Niuatahi south	Plume	-15.394	-174.002	1565	2.75	2.77	0.26	-/-/-
Ns-Site7-p3	73CTD_b11	Niuatahi south	Plume	-15.389	-173.994	1220	3.4	2.6	0.25	-/-/-
M-Site8-bp	119CTD_b1*	Maka_NELSC	Below plume	-15.422	-174.284	1450	2.9	2.76	0.32	-/-/+
M-Site8-p	119CTD_b4*	Maka_NELSC	Plume	-15.422	-174.284	1380	3.0	2.75	0.72	-/-/+
M-Site8-ap	119CTD_b16*	Maka_NELSC	Above plume	-15.422	-174.284	1250	3.2	2.8	0.25	-/-/-
Nn-1ROV-rp	103ROV_05	Niuatahi north	Rising plume	-15.327	-173.996	1675	Nd	Nd	Nd	-/-/-
Ns-2ROV-rp	110ROV_05	Niuatahi south	Rising plume	-15.3874	-173.998	1611	Nd	Nd	Nd	-/-/-

M-3ROV-rp	116ROV_18	Maka_NELSC	Rising plume	-15.4226	-174.284	1544	Nd	Nd	Nd	-/-/-
M-4ROV-rp	116ROV_14	Maka_NELSC	Rising plume	-15.422	-174.284	1537	Nd	Nd	Nd	-/-/-

*The cruise report (4) refers to this CTD cast either as 119CTD (first results) or 121CTD (station list).

Table S2: Helium and neon isotope data for samples collected at Niua South, Niua North, Niuatahi and Maka.

Sample characteristic	Sample	Real sample name	$^3He/^4He$	Ne/He	δ^3He	$\delta^{22}Ne$
Plume	NiuaS-Site1-p	06CTD_b9	1.950E-06	4.04E+00	40.93	0.12
Above plume	NiuaS-Site1-ap	06CTD_b13	1.739E-06	4.23E+00	25.65	0.14
Plume	NiuaN-Site3-p	35CTD_b2	6.619E-06	1.70E+00	378.24	0.90
Above plume	NiuaN-Site3-ap	35CTD_b12	2.403E-06	3.84E+00	73.66	0.16
Weak plume	Nn-Site5-wp	87CTD_b3	3.041E-06	3.59E+00	119.70	0.20
Plume	Nn-Site5-p	87CTD_b7	3.182E-06	3.49E+00	129.88	0.18
Above plume	Nn-Site5-ap	87CTD_b13	1.984E-06	4.13E+00	43.38	0.22
Below plume	Ns-Site6-bp	88CTD_b9	1.838E-06	4.22E+00	32.79	0.04
Rising plume	Nn-1ROV-rp	103ROV_05	4.554E-06	5.37E-01	229.03	-21.24
Rising plume	Ns-2ROV-rp	110ROV_05	3.749E-06	3.11E+00	170.86	0.30
Rising plume	M-3ROV-rp	116ROV_18	7.177E-06	1.98E+00	418.57	0.61
Rising plume	M-4ROV-rp	116ROV_14	5.050E-06	2.78E+00	264.91	0.13
Below plume	M-Site8-bp	119CTD_b1	6.331E-06	2.39E+00	357.45	0.30
Plume	M-Site8-p	119CTD_b4	8.438E-06	1.51E+00	509.71	0.94
Above plume	M-Site8-ap	119CTD_b16	2.119E-06	4.10E+00	53.10	0.23

Table S3: Sequencing and assembly statistics. Assembly statistics were assessed using Quast v5.0.2 (5).

Sample	Metatranscriptome		Metagenome			Metagenome Assembly				
	Raw Reads (2x150bp)		Raw Reads (2x250bp)	Average read length [bp]	Coverage [%]	N50	GC [%]	Number of contigs > 5000 bp	number of contigs	largest contig [bp]
Nn-Site5-wp	3.67E+07	4.66E+07	3.17E+07	250	97	4369	51.7	2371	25029	825579
Ns-Site6-bp	3.79E+07	3.81E+07	3.29E+07	250	86	2327	48.1	4467	86785	1403042

Table S4: The statistics of MAGs retrieved in this study. MAGs were retrieved from single assemblies of Nn-Site5-wp and Ns-Site6-bp using CONCOCT (6) and manually refined in Anvi'o v6.1 (7). Quality was assessed using CheckM (8).

MAG	Taxonomy	Comp. [%]	Cont.	Length [bp]	GC [%]	Number of contigs	N50
Alc-1	*Alcanivorax*	99.0	1.5	3586158	64.8	123	93656
Alc-2	*Alcanivorax*	98.8	0.6	3533977	64.8	43	398266
Alc-3	*Alcanivorax*	99.8	0.8	3746458	58.3	107	103619
Alc-4	*Alcanivorax*	89.4	5.7	3230525	58.6	478	9015
Alc-5	*Alcanivorax*	94.4	2.8	3646770	58.0	184	31020
Alc-6	*Alcanivorax*	92.2	2.6	2930344	58.9	676	5624
SUP05-7	SUP05	84.4	6.0	1060180	38.2	267	5126
SUP05-8	SUP05	76.24	2.1	1301652	37.5	356	4351
MAG-9	*Sulfitobacter*	99.4	0.7	3745460	60.4	109868	53
MAG-10	*Alteromonas*	72.0	6.0	4463290	44.3	882	6038
MAG-11	*Marinobacter*	57.8	1.5	1934139	51.5	421	5329
MAG-12	*Flavobacteriales*	99.6	1.6	3052595	33.7	65	97240
MAG-13	*Thiomicrospira*	95.2	1.6	1643760	34.9	186	12456
MAG-14	*Acidobacteria*	60.6	4.3	2808735	66.2	1454	2053
MAG-15	*Erythrobacter*	78.8	1.8	2522269	64.5	853	3457

Table S5: Datasets searched for *Alcanivorax* presence. These datasets were recruited onto retrieved MAGs with 99% identity threshold using BBMap (18).

Environment	BioProject	Dataset	Reference
TARA Oceans Database	PRJEB1787	Metagenome	(9)
Malaspina Expedition		Metagenome	(10)
Malaspina Expedition - 4x		Metatranscriptome	(10)
Stimulated oil spill	PRJNA320927	Metagenome	(11)
Deepwater Horizon Oil Spill	PRJNA336904	Metagenome	x
Mariana trench	PRJNA421240	16S rRNA amplicon	(12)
Hydrocarbon-rich deep sea sediments in MAR (Juan de Fuca Ridge and Guaymas Basin)	PRJNA485566	16S rRNA amplicon	x
Methane seeps along the Pacific margin	PRJNA315164	16S rRNA amplicon	(13)
Hydrothermal vent plumes of Guaymas Basin	PRJNA72707	Metatranscriptome	(14)
Hydrothermal vent plumes of Guaymas Basin	PRJNA77837	Metagenome	(14)
Hydrothermal vent plumes of Brothers	PRJEB42974	Metagenome	(15)
Hydrothermal vent plumes of Macauley	PRJEB42974	Metagenome	(15)
Hydrothermal vent plumes of Lau Basin	PRJNA234377	Metagenome	(16)
Hydrothermal vent plumes of Woody Crack	PRJEB11362	Metagenome	(17)

Table S6: Transcription of *Alcanivorax* Alc-2 and Alc-3 in the Malaspina metatranscriptome. Samples correspond to Malaspina metagenomes, where *Alcanivorax* were highly abundant.

	Latitude	Longitude	Depth	Alc-2 RPKM	Alc-3 RPKM	NCBI Biosample Accession
MP0747	-32.4846	12.4609	3904	0.025	0.03	SAMN06268824
MP1089	-29.81	82.62	-	0.003	0.05	SAMN07687584
MP0441	-22.5716	-36.5529	3918	0.26	0.128	SAMN06268822
MP1372	-39.148	135.0813	4000	0.009	0.3	SAMN06268829

References

1. Sültenfuß J, Roether W, Rhein M. The Bremen mass spectrometric facility for the measurement of helium isotopes, neon, and tritium in water. Isot Environ Health Stud. 2009;45(2):83-95.

2. Neuholz R, Kleint C, Schnetger B, Koschinsky A, Laan P, Middag R, Sander S, Thal J, Türke A, Walter M, Zitoun R. Submarine hydrothermal discharge and fluxes of dissolved Fe and Mn, and He isotopes at Brothers Volcano based on radium isotopes. Minerals. 2020;10(11):969.

3. Klose L, Kleint C, Türke A, Haase KM (2022): Raw data of physical oceanography during RV SONNE cruise SO263. PANGAEA, https://doi.org/10.1594/PANGAEA.949874

4. Haase KM, Beier C, Bach W, Kleint C, Anderson MO, Rubin K, et al. SO-263 Cruise Report: Tonga Rift. 2018.

5. Gurevich A, Saveliev V, Vyahhi N, Tesler G. QUAST: Quality assessment tool for genome assemblies. Bioinformatics. 2013;29(8):1072-5.

6. Alneberg J, Bjarnason BS, De Bruijn I, Schirmer M, Quick J, Ijaz UZ, et al. Binning metagenomic contigs by coverage and composition. Nat Methods. 2014;11(11):1144-6.

7. Eren AM, Kiefl E, Shaiber A, Veseli I, Miller SE, Schechter MS, et al. Community-led, integrated, reproducible multi-omics with anvi'o [Internet]. Vol. 6, Nat Microbiol. 2021;6(1): 3–6.

8. Parks DH, Imelfort M, Skennerton CT, Hugenholtz P, Tyson GW. CheckM: Assessing the quality of microbial genomes recovered from isolates, single cells, and metagenomes. Genome Res. 2015;25(7):1043–55.

9. Guidi L, Chaffron S, Bittner L, Eveillard D, Larhlimi A, Roux S, et al. Plankton networks driving carbon export in the oligotrophic ocean. Nature. 2016;532(7600):465-70.

10. Duarte CM. Seafaring in the 21st century: The Malaspina 2010 circumnavigation expedition. Limnol Oceanogr Bull. 2015;24:11–14.

11. Hu P, Dubinsky EA, Probst AJ, Wang J, Sieber CM, Tom LM, Gardinali PR, Banfield JF, Atlas RM, Andersen GL. Simulation of Deepwater Horizon oil plume reveals substrate specialization within a complex community of hydrocarbon degraders. Proc Natl Acad Sci. 2017;114(28):7432-7.

12. Li WL, Huang JM, Zhang PW, Cui GJ, Wei ZF, Wu YZ, et al. Periodic and spatial spreading of alkanes and Alcanivorax bacteria in deep waters of the Mariana Trench. Appl Environ Microbiol. 2019;85(3):e02089-18.

13. Pasulka AL, Goffredi SK, Tavormina PL, Dawson KS, Levin LA, Rouse GW, Orphan VJ. Colonial tube-dwelling ciliates influence methane cycling and microbial diversity within methane seep ecosystems. Front Mar Sci. 2017:276.

14. Sheik CS, Jain S, Dick GJ. Metabolic flexibility of enigmatic SAR324 revealed through metagenomics and metatranscriptomics. Environ Microbiol. 2014;16(1):304-17.

15. Dede B, Hansen CT, Neuholz R, Schnetger B, Kleint C, Walker S, et al. Niche differentiation of sulfur-oxidizing bacteria (SUP05) in submarine hydrothermal plumes. ISME J. 2022;16(6):1479-90.

16. Anantharaman K, Breier JA, Dick GJ. Metagenomic resolution of microbial functions in deep-sea hydrothermal plumes across the Eastern Lau Spreading Center. ISME J. 2016;10(1):225–39.

17. Meier D V., Bach W, Girguis PR, Gruber-Vodicka HR, Reeves EP, Richter M, et al. Heterotrophic Proteobacteria in the vicinity of diffuse hydrothermal venting. Environ Microbiol. 2016;18(12):4348–68.

18. Bushnell B. BBMap (version 35.14). 2015. Available at https://sourceforge.net/projects/bbmap/

19. Karthikeyan S, Rodriguez-R LM, Heritier-Robbins P, Hatt JK, Huettel M, Kostka JE, et al. Genome Repository of Oiled Systems (GROS): An interactive and searchable database that expands the catalogued diversity of crude oil-associated microbes. bioRxiv. 2019;838573.

20. Callahan BJ, McMurdie PJ, Rosen MJ, Han AW, Johnson AJA, Holmes SP. DADA2: High-resolution sample inference from Illumina amplicon data. Nat Methods. 2016;13(7):581-3.

21. Diehl A, Bach W. MARHYS (MARine HYdrothermal Solutions) Database: A global compilation of marine hydrothermal vent fluid, end member, and seawater compositions. Geochemistry, Geophys Geosystems. 2020;21(12) :e2020GC009385.

22. Kopylova E, Noé L, Touzet H. SortMeRNA: Fast and accurate filtering of ribosomal RNAs in metatranscriptomic data. Bioinformatics. 2012;28(24):3211-7.

23 . Quast C, Pruesse E, Yilmaz P, Gerken J, Schweer T, Yarza P, et al. The SILVA ribosomal RNA gene database project: Improved data processing and web-based tools. Nucleic Acids Res. 2013;41(D1):D590-6.

24. Priest T, Heins A, Harder J, Amann R, Fuchs BM. Niche partitioning of the ubiquitous and ecologically relevant NS5 marine group. ISME J. 2022;1–13.

25. Arndt D, Grant JR, Marcu A, Sajed T, Pon A, Liang Y, et al. PHASTER: a better, faster version of the PHAST phage search tool. Nucleic Acids Res. 2016;44(W1):W16-21.

26. Menzel P, Ng KL, Krogh A. Fast and sensitive taxonomic classification for metagenomics

with Kaiju. Nat Commun. 2016;7(1):1-9.

Supplementary table captions

Table S1: Samples analyzed in this study. Given are the sampling location, determined physical and chemical parameters (1), and the experiments conducted on the different samples during this study (MG: Metagenomics, MT: Metatranscriptomics, FISH: fluorescence *in situ* hybridization). Nd- not determined.

Table S2: Helium and neon isotope data for samples collected at Niua South, Niua North, Niuatahi and Maka.

Table S3: Sequencing and assembly statistics. Assembly statistics were assessed using Quast v5.0.2 (5).

Table S4: The statistics of MAGs retrieved in this study. MAGs were retrieved from single assemblies of Nn-Site5-wp and Ns-Site6-bp using CONCOCT (6) and manually refined in anvi'o v6.1 (7). Quality was assessed using CheckM (8).

Table S5: Datasets searched for *Alcanivorax* presence. These datasets were recruited onto retrieved MAGs with 99% identity threshold using BBMap (18).

Table S6: Transcription of *Alcanivorax* Alc-2 and Alc-3 in the Malaspina metatranscriptome. Samples correspond to Malaspina metagenomes, where *Alcanivorax* were highly abundant.

Supplementary figure captions

Figure S1. Overview of hydrothermal systems sampled in this study. A) Geographic location of the Tonga Arc and the area of interest. B) Bathymetric map of four sampled volcanoes, Niua North, Niua South, Niuatahi and Maka (Northeast Lau Spreading Center). The sampling sites are depicted with a star. The Site names represent CTD and ROV dives. Bathymetric data are taken from GEBCO Bathymetric Compilation Group (2020). C) Photos taken at four volcanoes. Photos were taken during ROV dives (MARUM QUEST 4000) and are copyright of MARUM - Center for Marine Environmental Sciences, University of Bremen.

Figure S2. Oxygen, turbidity and potential temperature of station A) B-Site (01CTD), B) NiuaS-Site1 (06CTD) and C) Ns-Site6 (88CTD). Sensors such as SBE 43, ITS-90 and WET Labs ECO measured oxygen, temperature and turbidity, respectively. Data was analysed using Seasoft V2: SBE data processing.

Figure S3. Workflow of sampling procedure onboard of the ship. Niskin bottles 1 and 2 represent different samples. Created with Biorender.com.

Figure S4. Linear correlation between TAD80 (Truncated Average sequencing Depth) (19) and RPKM of MAGs in 20 Malaspina metagenomes. Grey area represent zero TAD80 values, which are used as a threshold to determine absence of a MAG in a metagenome.

Figure S5. Phylogenetic tree of *Alcanivorax* spp. based on 16S rRNA gene sequences. This tree is a consensus tree calculated based on 30 long sequences using PhyML, a 30% position conservation filter. ASVs and a 16S rRNA gene extracted from Alc-3 are depicted in blue. On the right hand side the target subgroups of *Alcanivorax*-specific probes (ALV461, ALV735 and ALV735-b) are indicated.

Figure S6. Relative read abundance of *Alcanivorax* ASVs in Niuatahi and Maka volcanoes. ASVs were analysed using DADA2 (20). All other ASVs which have less than 1% abundance are shown in gray.

Figure S7. Non-metric multidimensional scaling analysis (NMDS) calculated using Bray-Curtis dissimilarity matrix based on the ASVs. Three categories including background, plume and the rising plume are colored black, light blue and green, respectively. Hclust cut-off was 0.45.

Figure S8. Principal component analysis (PCA) calculated using H_2, CH_4 and H_2S concentrations of 232 fluids expelled at hydrothermal vents. Samples are grouped based on geological

characteristics of vent fields: BAB (Back Arc Basins), MOR (Mid-Ocean Ridge), Volcano (Volcanic arcs or Intra-plate volcanoes) and low temperature fluid or white smokers. Chemical data were taken from MARHYS v1 database (21). Chemical data of Niuatahi, Maka, Niua North and South were taken from the Cruise Report SO263 (4).

Figure S9. Total cell counts and relative abundance of selected microbial taxa. Total cell counts were determined by counting DAPI stained cells, whereas specific taxonomic clades were targeted with specific probes and counted. The abundance of the microbial groups was counted relative to DAPI stained cells.

Figure S10. Relative abundance of 16S rRNA genes extracted from metagenomes (MG) and metatranscriptomes (MT). The two metatranscriptomes were technical duplicates (R1 and R2). 16S rRNA reads were extracted from metagenomes and metatranscriptomes using SortMeRNA (22) and classified using SilvaNGS v138 (23).

Figure S11. Average amino acid identity (AAI) of *Alcanivorax* MAGs retrieved in this study and their closely-related cultivated species and high and intermediate quality MAGs from GROS (19).

Figure S12. Relative abundance of *Alcanivorax* MAGs in two metagenomes. Metagenomics raw reads were recruited on *Alcanivorax* MAGs using BBMap (18) (minimum identity 99%). Abundance is given in reads per kilobase per million (RPKM).

Figure S13. The pangenome of the *Alcanivorax* genus using *Alcanivorax* MAGs retrieved in this study and genomes of the most studied cultivated species including, *Alcanivorax borkumensis*, *Alcanivorax hongdensis*, *Alcanivorax jadensis, Alcanivorax profundi*, *Alcanivorax* strain DG881, *Alcanivorax* strain VBW001 and *Alcanivorax venustensis*. Black lines depict the occurrence of gene clusters. The “Core genome” section corresponds to the genes shared between all genomes. Group 1 corresponds to genes shared between all genomes but *Alcanivorax venustensis* and the closely-related MAGs, Alc-2 and Alc-1. Genes corresponding only to these three MAGs but not to other genomes, were grouped in Group 2. SCG clusters refer to single-copy gene clusters. The right-hand side section provides additional information on hierarchical clustering of the MAGs based on single-copy genes.

Figure S14. Comparison of peptidases and transporters gene per Mbp in *Alcanivorax* genomes. Peptidases and transporters were annotated as described in Priest et al. (24).

Figure S15. Heatmap of the resistance genes in *Alcanivorax* MAGs retrieved in this study and their closely-related cultivated species including *Alcanivorax* HI0044, *Alcanivorax venustensis*, *Alcanivorax* DG881, *Alcanivorax jadensis* and *Alcanivorax* VBW004. The legend represents the number of resistance genes in each MAG.

Figure S16. Mercuric reductase gene placement between transposases in a) Alc-1 and b) Alc-2. **T**he genes are denoted by black arrows and the two replicates of each metatranscriptome (Nn-Site5-wp and Nn-Site6-bp) were mapped to them (identity=97%). The expression is denoted in four lanes of arrows beneath genes and their color indicates the transcript per million (TPM).

Figure S17. Placement of additional FAD-binding monooxygenase gene between transposases in Alc-3. The genes are denoted by black arrows. Metatranscriptomes of sample Nn-Site5-wp and Nn-Site6-bp (2x technical replicates per sample) were mapped to Alc-3 with a minimum identity of 97%. The expression is denoted in four lanes and their color indicates the transcript per million (TPM).

Figure S18. Heatmap of the transporters in *Alcanivorax* MAGs retrieved in this study and their closely-related cultivated species *Alcanivorax* HI004, *Alcanivorax venustensis*, *Alcanivorax* DG881, *Alcanivorax jadensis* and *Alcanivorax* VBW004. Legend represents the number of genes in each MAG.

Figure S19. Viral sequences in Alc-3 analyzed using PHASTER (25). Both sequences had >90 score. Viral genes are depicted in different colors.

Figure S20. Expression of genes involved in biosurfactant synthesis. Genes analysed are *plsC* – Acetyltransferase domain protein, *plsB* – Glycerol-3-phosphate acyltransferase, *plsY* – Probable glycerol-3-phosphate acyltransferase, *lolD* – Lipoprotein.releasing system ATP-binding protein, *psd* – Phosphatidylserine decarboxylase. Transcripts were normalized to the length of the gene and total number of reads in metatranscriptoms (TPM).

Figure S21. Abundance of A) Alc-3 and B) Alc-6 in Malaspina dataset (10). Reads were mapped unambiguously using BBmap (18) with 99% minimum identity. Abundance was calculated as RPKM.

Figure S22. Abundance of *Alcanivorax* MAGs in other plumes and oil spill metagenomes. A) RPKM of MAGs in plumes of the Lau Basin as well as Brothers and Macauley volcano in the Kermadec arc. Sample 49CTD_b16 is represented on the secondary axes. B) RPKM of MAGs in stimulated and real oil spill systems and alkane-rich samples of Mariana Trench.

Figure S23. Comparison of *Alcanivorax* relative abundance between different cultivation-independent techniques. Raw read recruitment represent the fraction of metagenomics raw reads recruited on the *Alcanivorax* MAGs. 16S RNA was sorted from metagenomes and metatranscriptomes using SortMeRNA (19). Cells were visualized and counted using CARD-FISH with a mixture of ALV735 and ALV735-b probes. All metagenomics reads were taxonomically affiliated using Kaiju (26).
