# Supplementary material for: High abundance of hydrocarbon-degrading Alcanivorax in plumes of hydrothermally active volcanoes in the South Pacific Ocean
Source: ISME J. 2023 Jan 31;17(4):600–10. doi: 10.1038/s41396-023-01366-4 (PMC10030979; doi:10.1038/s41396-023-01366-4)
Supplement: Supplementary file 2 — Supplementary Figures [file 41396_2023_1366_MOESM2_ESM.pdf]

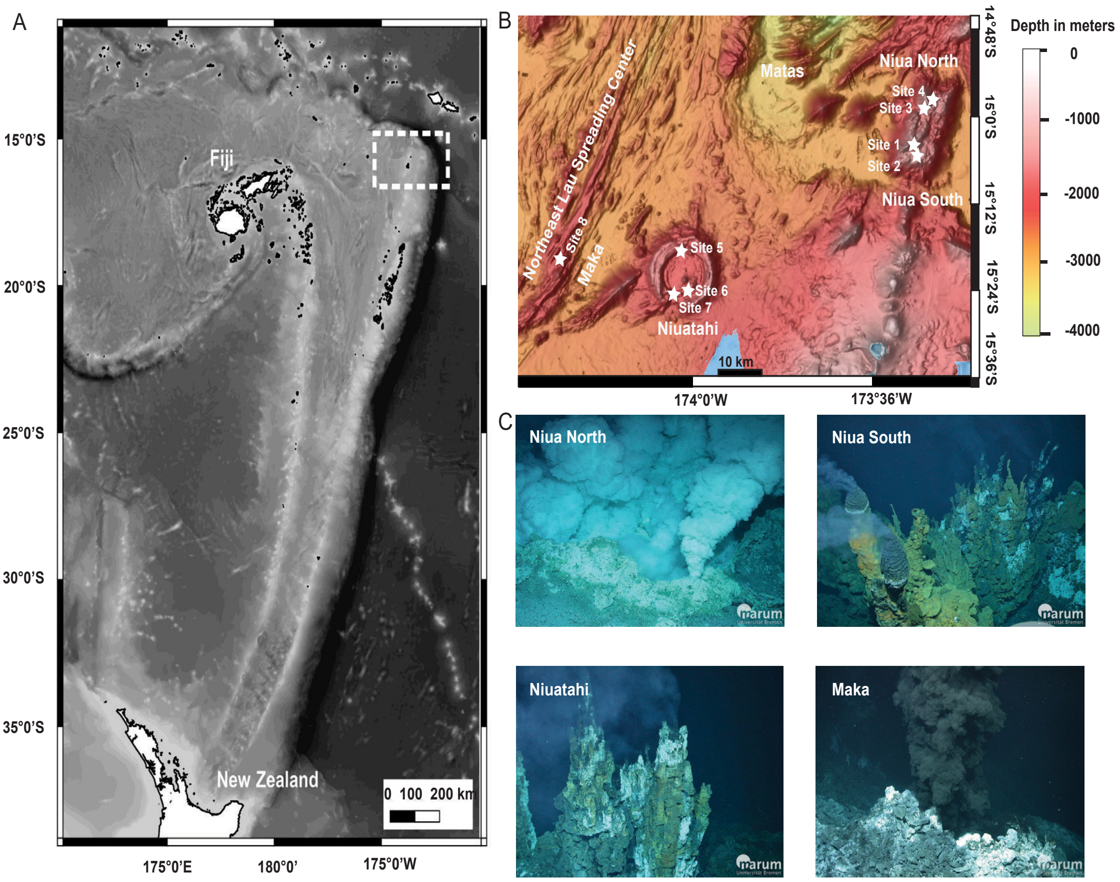

Figure S1 Overview of hydrothermal systems sampled in this study. A) Geographic location of the Tonga Arc and the area of interest. B) Bathymetric map of four sampled volcanoes, Niua North, Niua South, Niuatahi and Maka (Northeast Lau Spreading Center). The sampling sites are depicted with a star. The Site names represent CTD and ROV dives. Bathymetric data are taken from GEBCO Bathymetric Compilation Group (2020). C) Photos taken at four volcanoes. Photos were taken during ROV dives (MARUM QUEST 4000) and are copyright of MARUM - Center for Marine Environmental Sciences, University of Bremen.

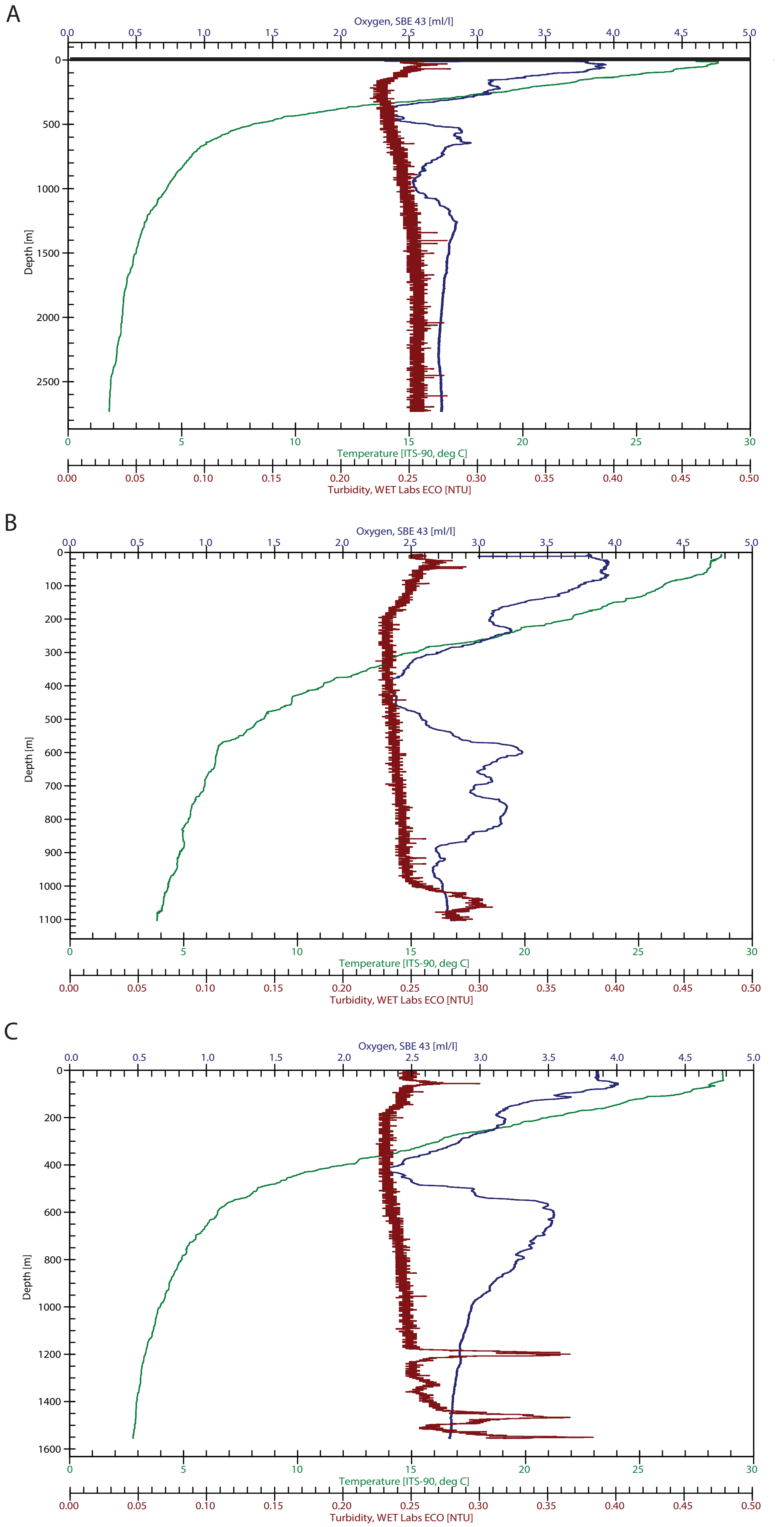

Figure S2. Oxygen, turbidity and potential temperature of station A) B-Site (01CTD), B) NiuaS-Site1 (06CTD) and C) Ns-Site6 (88CTD). Sensors such as SBE 43, ITS-90 and WET Labs ECO measured oxygen, temperature and turbidity, respectively. Data was analysed using Seasoft V2: SBE data processing.

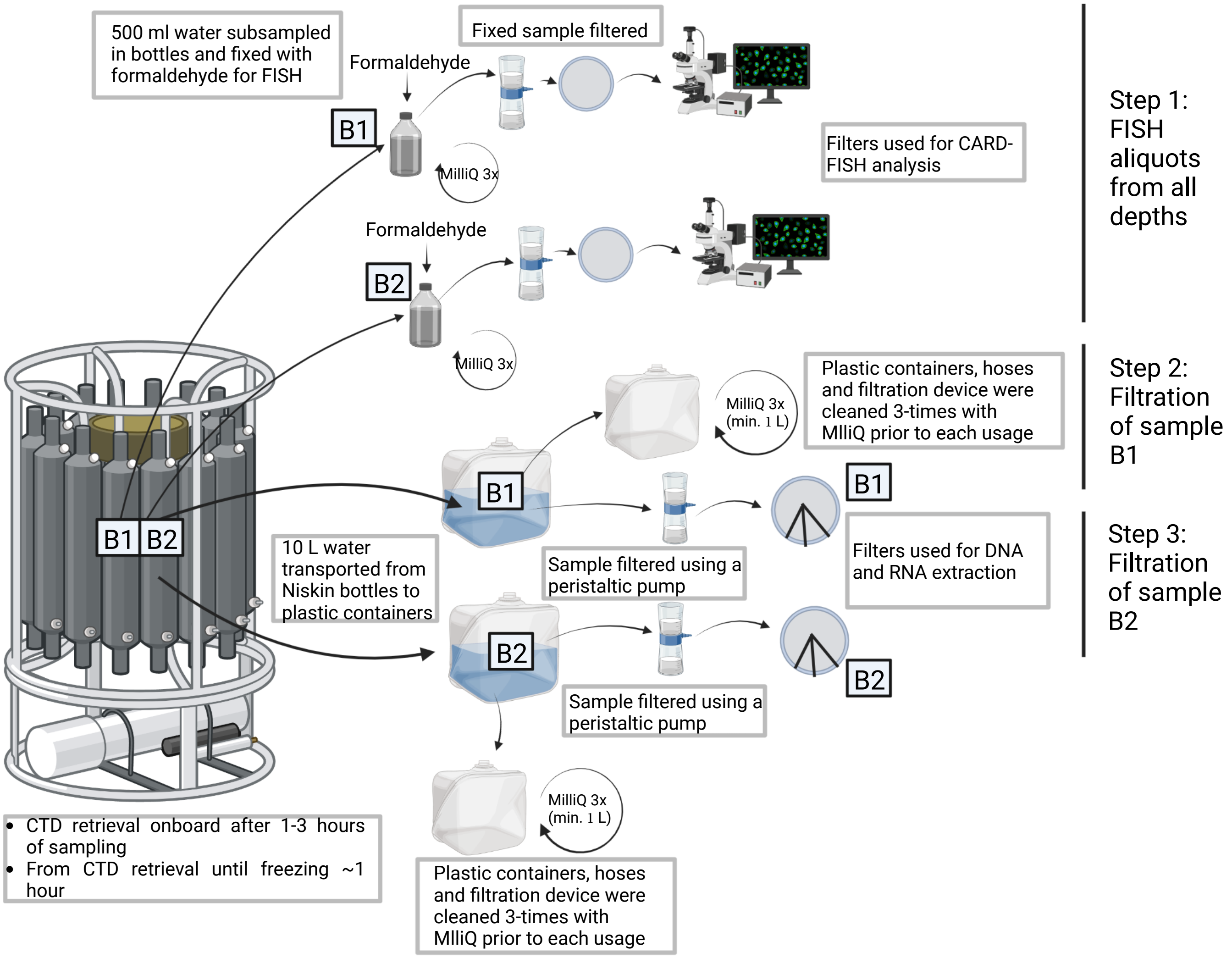

Figure S3. Workflow of sampling procedure onboard of the ship. Niskin bottles 1 and 2 represent different samples. Created with Biorender.com.

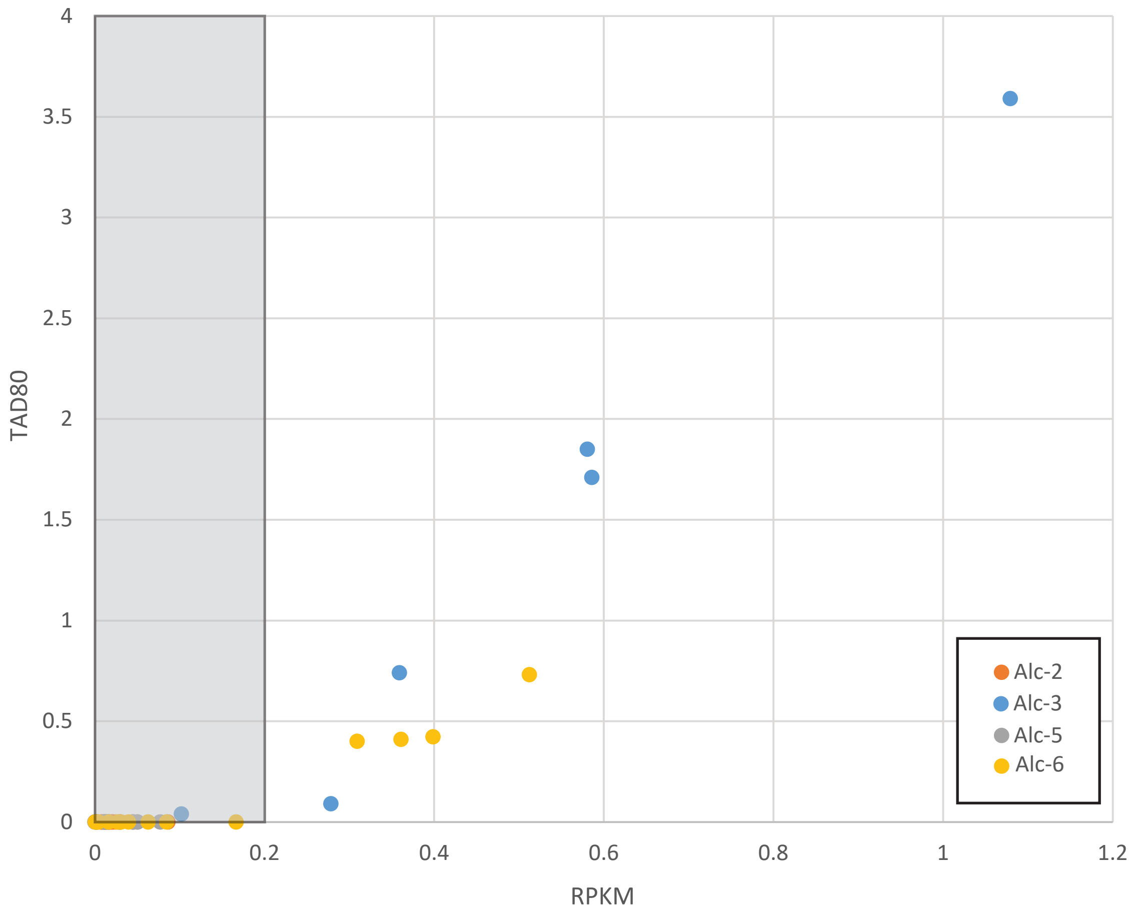

Figure S4. Linear correlation between TAD80 (Truncated Average sequencing Depth) (19) and RPKM of MAGs in 20 Malaspina metagenomes. Grey area represent zero TAD80 values, which are used as a threshold to determine absence of a MAG in a metagenome.

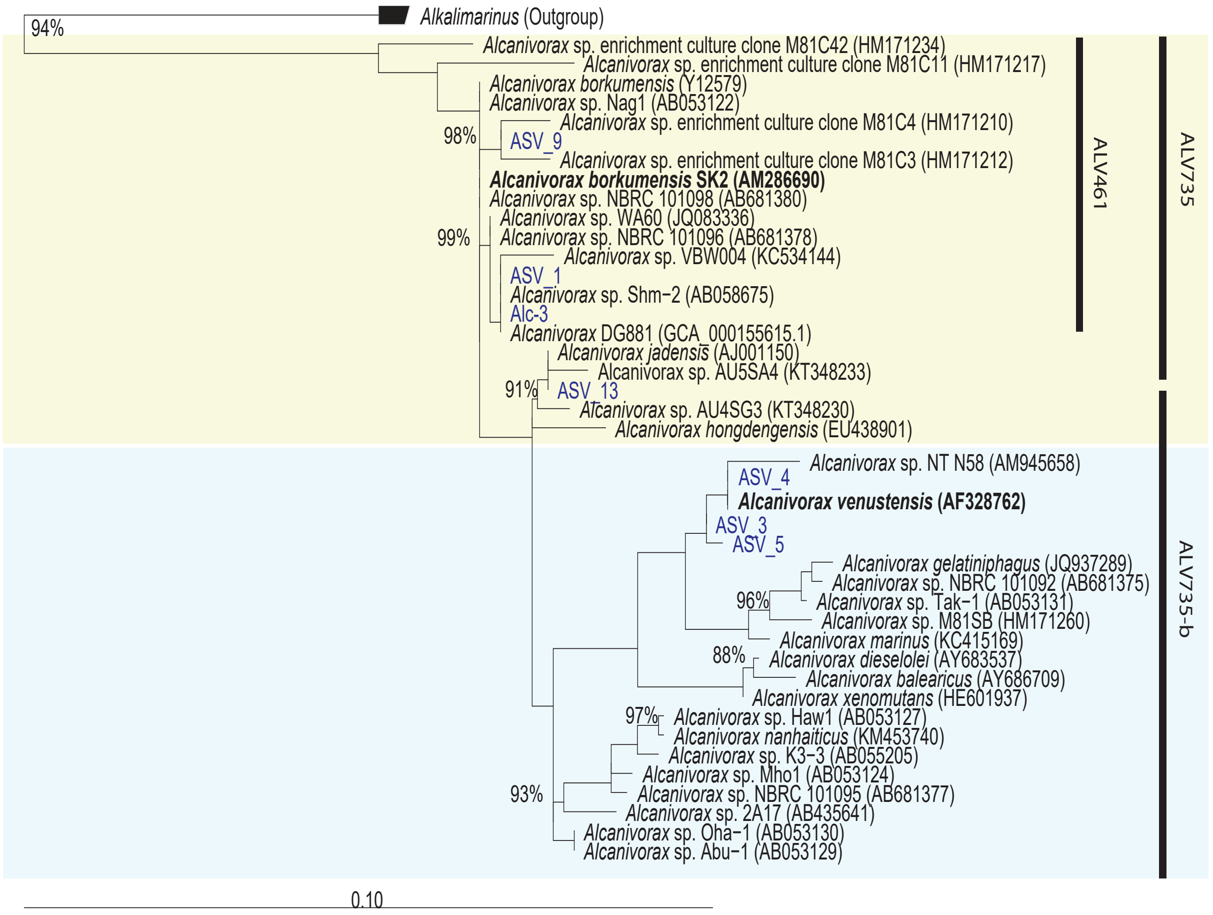

Figure S5. Phylogenetic tree of *Alcanivorax* spp. based on 16S rRNA gene sequences. This tree is a consensus tree calculated based on 30 long sequences using PhyML, a 30% position conservation filter. ASVs and a 16S rRNA gene extracted from Alc-3 are depicted in blue. On the right hand side the target subgroups of *Alcanivorax*-specific probes (ALV461, ALV735 and ALV735-b) are indicated.

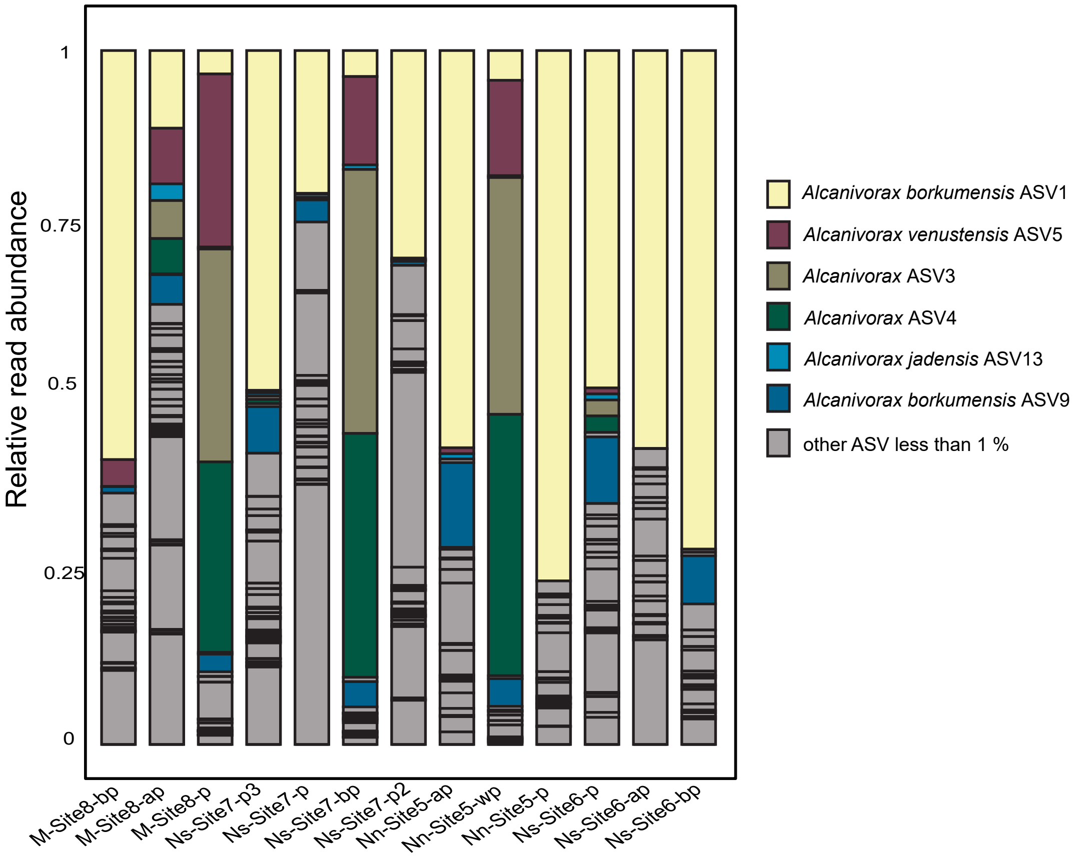

Figure S6. Relative read abundance of *Alcanivorax* ASVs in Niuatahi and Maka volcanoes. ASVs were analysed using DADA2 (20). All other ASVs which have less than 1% abundance are shown in gray.

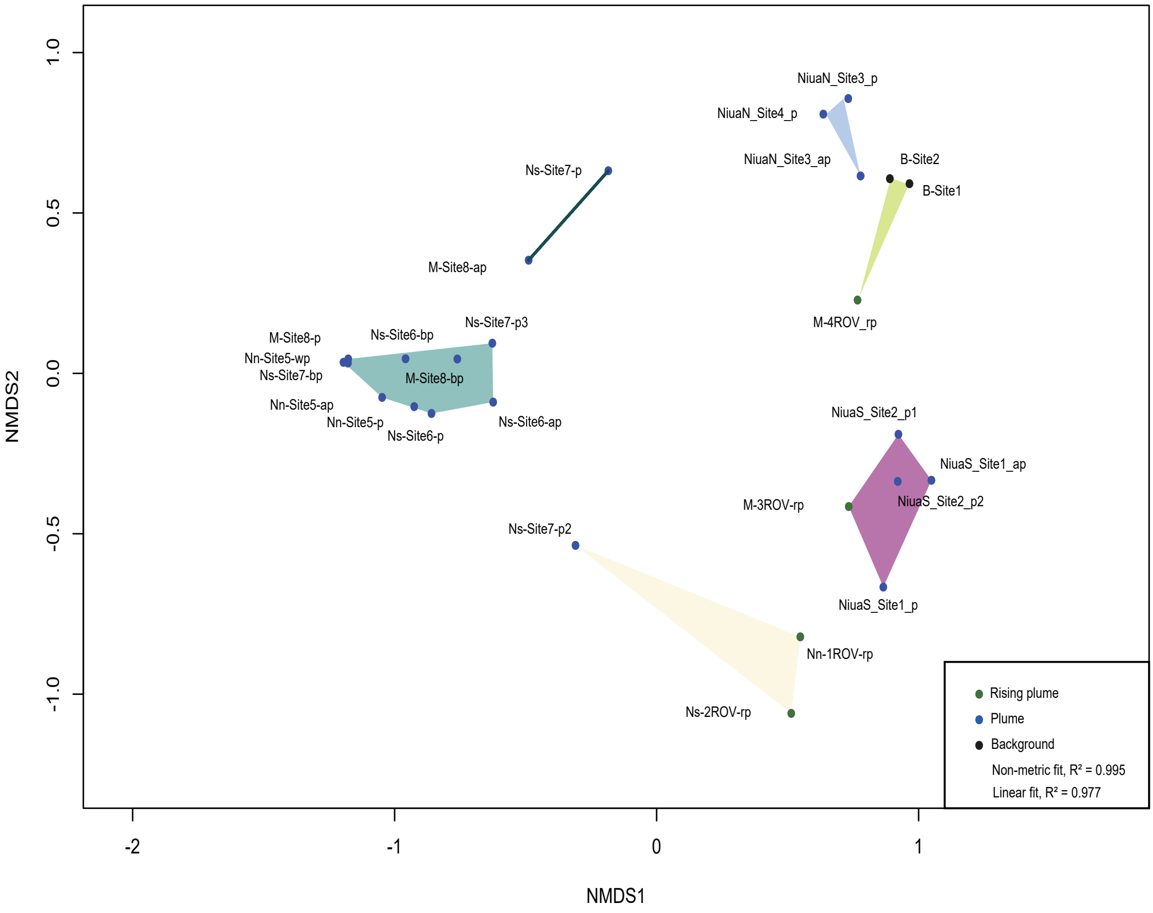

Figure S7. Non-metric multidimensional scaling analysis (NMDS) calculated using Bray-Curtis dissimilarity matrix based on the ASVs. Three categories including background, plume and the rising plume are colored black, light blue and green, respectively. Hclust cut-off was 0.45.

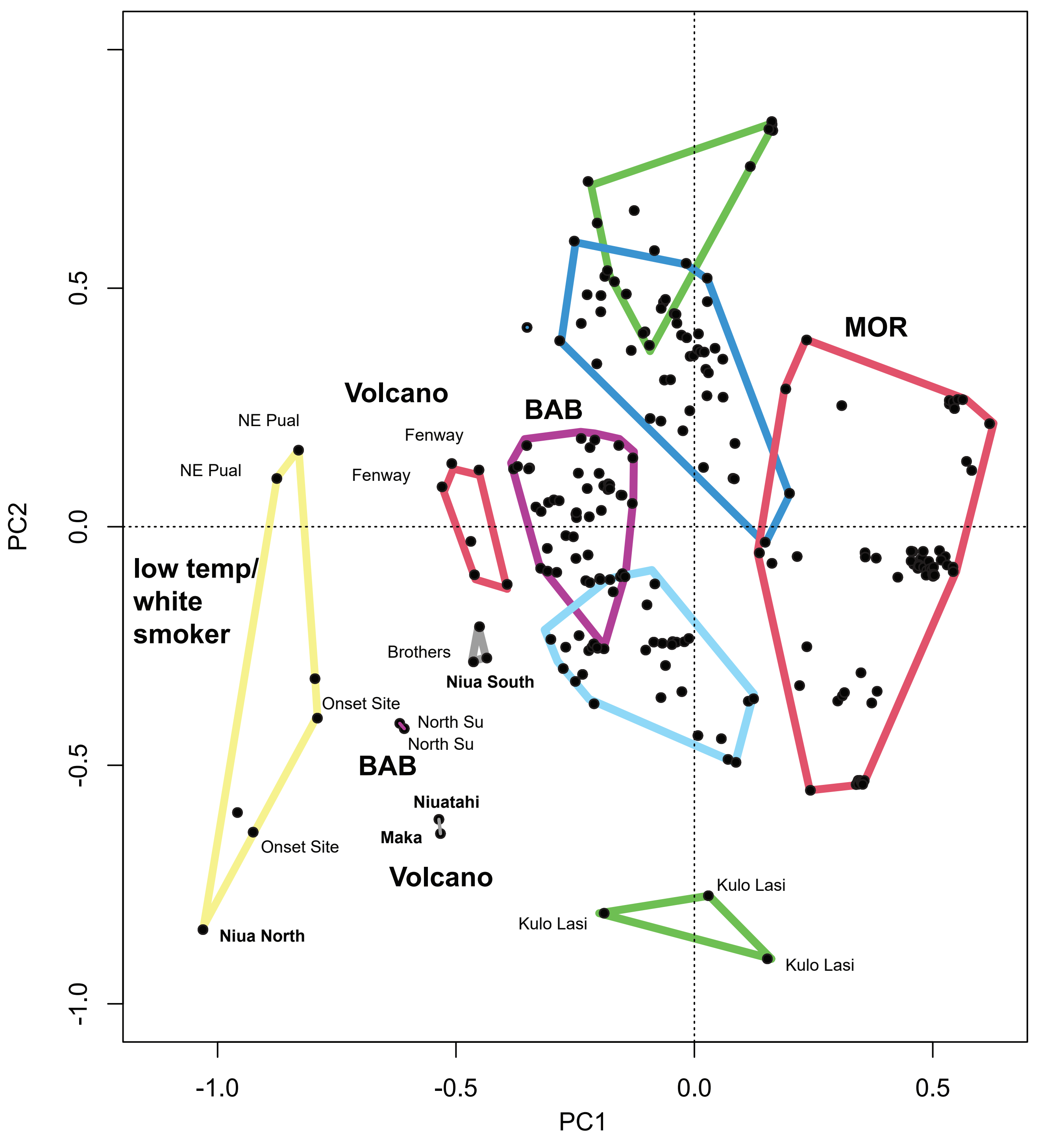

Figure S8. Principal component analysis (PCA) calculated using H_2, CH_4 and H_2S concentrations of 232 fluids expelled at hydrothermal vents. Samples are grouped based on geological characteristics of vent fields: BAB (Back-Arc Basins), MOR (Mid-Ocean Ridge), Volcano (Volcanic arcs or Intra-plate volcanoes) and low temperature fluid or white smokers. Chemical data were taken from MARHYS v1 database (21). Chemical data of Niuatahi, Maka, Niua North and South were taken from the Cruise Report SO263 (4).

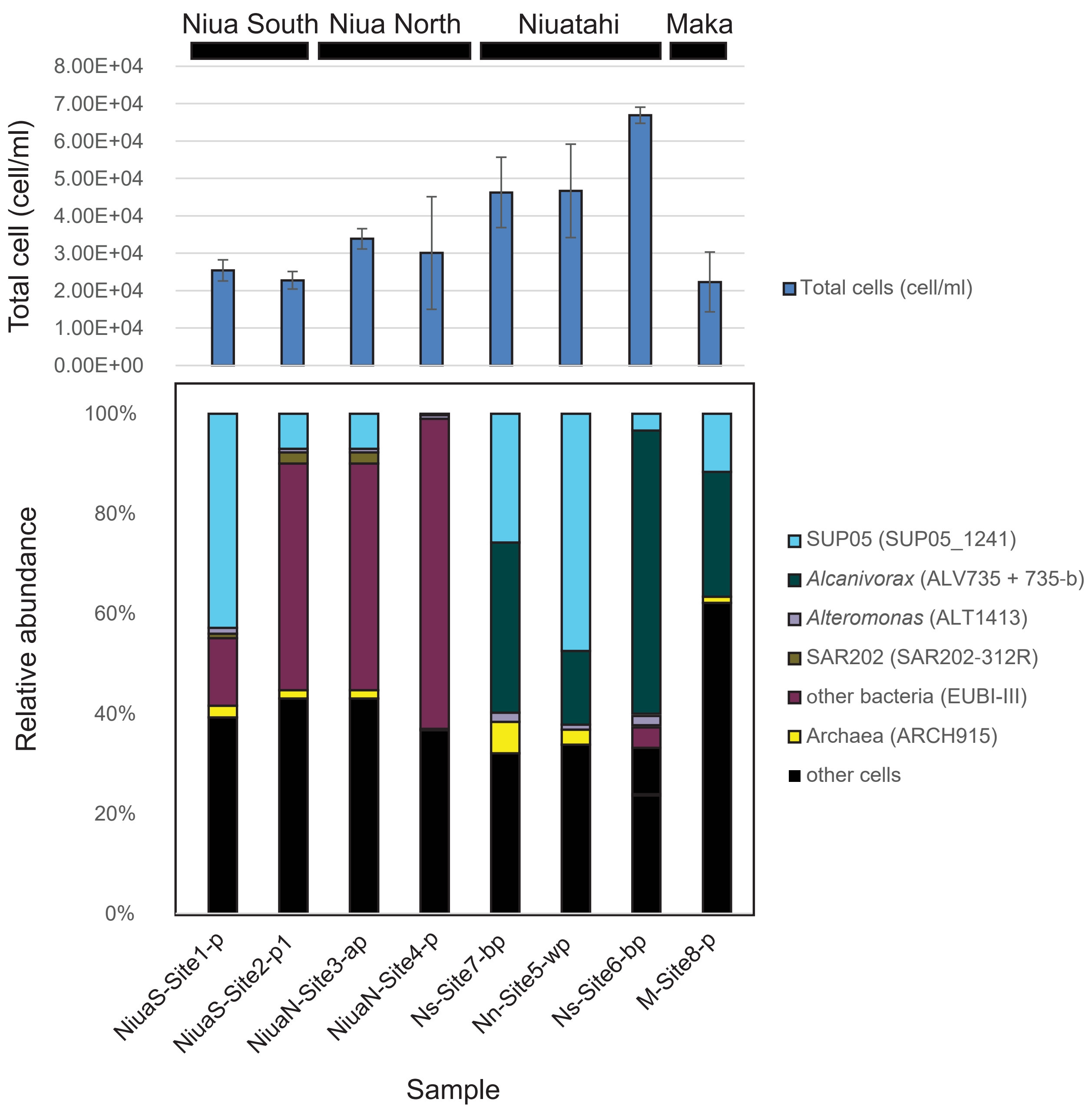

Figure S9. Total cell counts and relative abundance of selected microbial taxa. Total cell counts were determined by counting DAPI stained cells, whereas specific taxonomic clades were targeted with specific probes and counted. The abundance of the microbial groups was counted relative to DAPI stained cells.

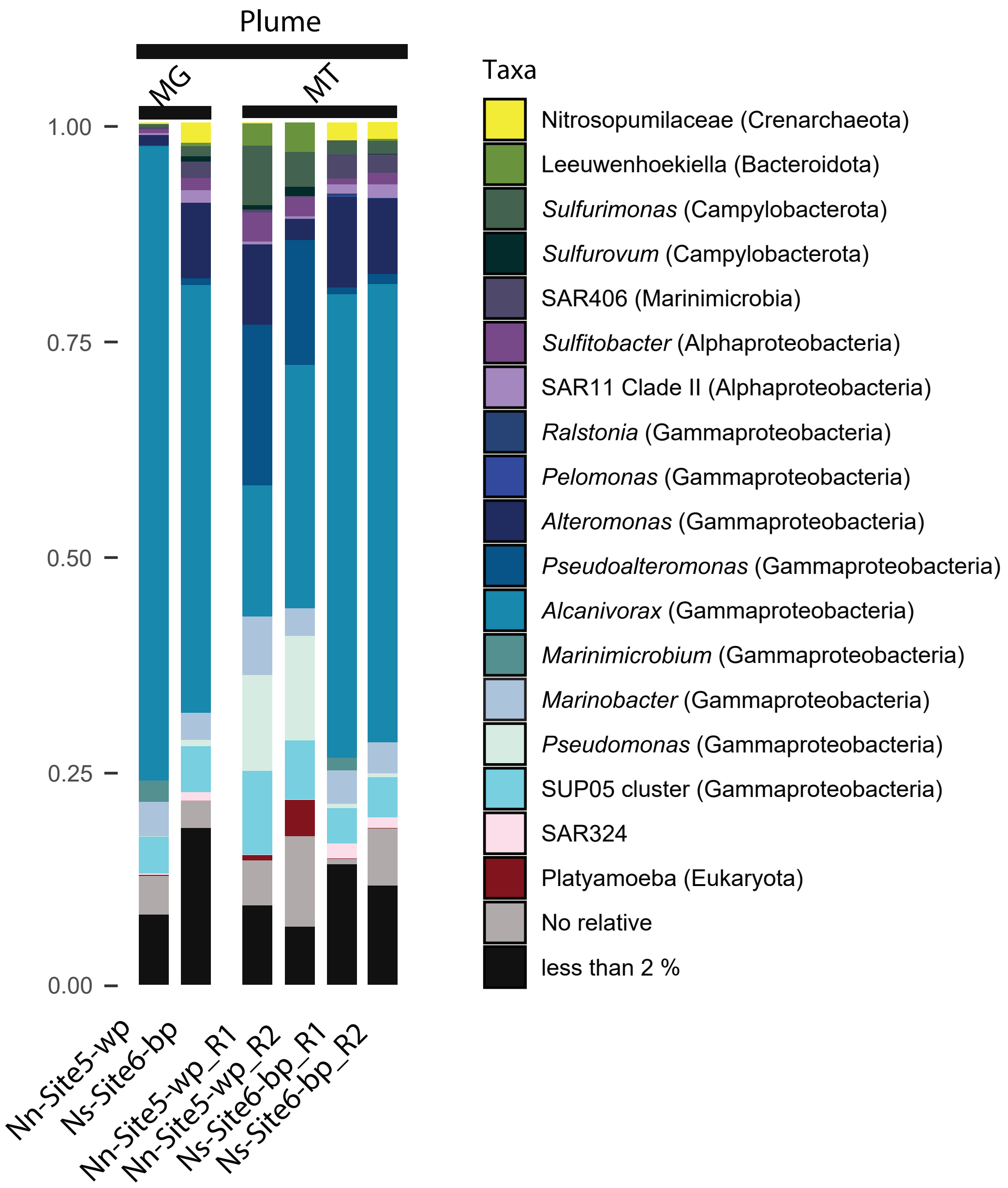

Figure S10. Relative abundance of 16S rRNA genes extracted from metagenomes (MG) and metatranscriptomes (MT). The two metatranscriptomes were technical duplicates (R1 and R2). 16S rRNA reads were extracted from metagenomes and metatranscriptomes using SortMeRNA (22) and classified using SilvaNGS v138 (23).

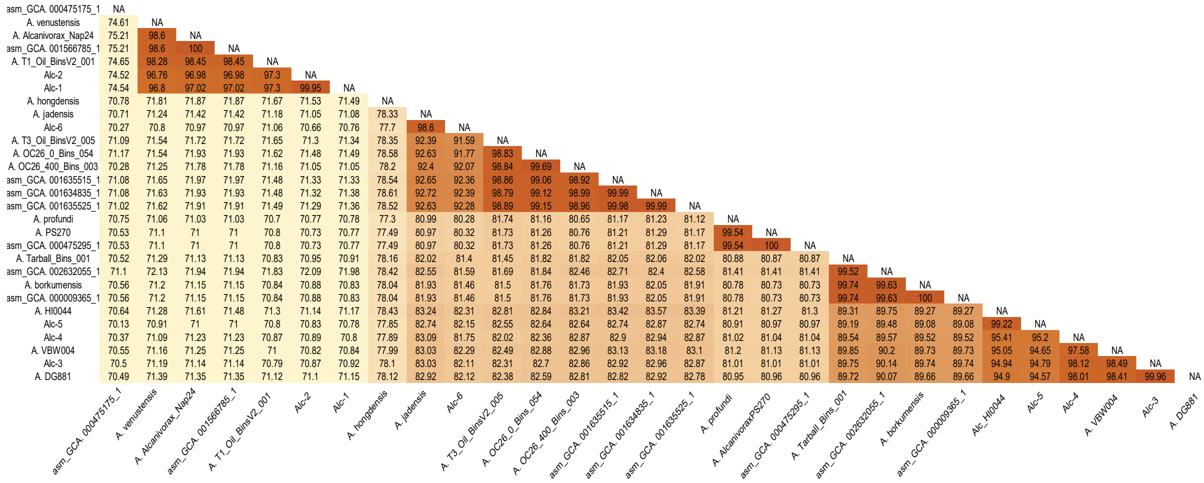

Figure S11. Average amino acid identity (AAI) of *Alcanivorax* MAGs retrieved in this study and their closely-related cultivated species and high and intermediate quality MAGs from GROS (19).

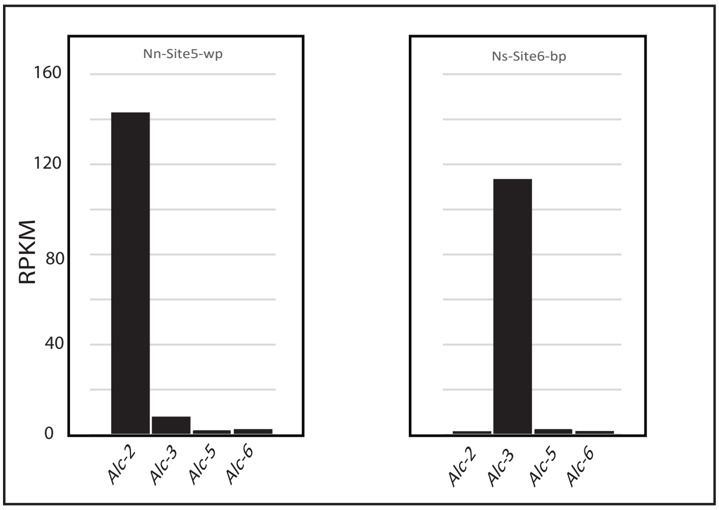

Figure S12. Relative abundance of *Alcanivorax* MAGs in two metagenomes. Metagenomics raw reads were recruited on *Alcanivorax* MAGs using BBMap (18) (minimum identity 99%). Abundance is given in reads per kilobase per million (RPKM).

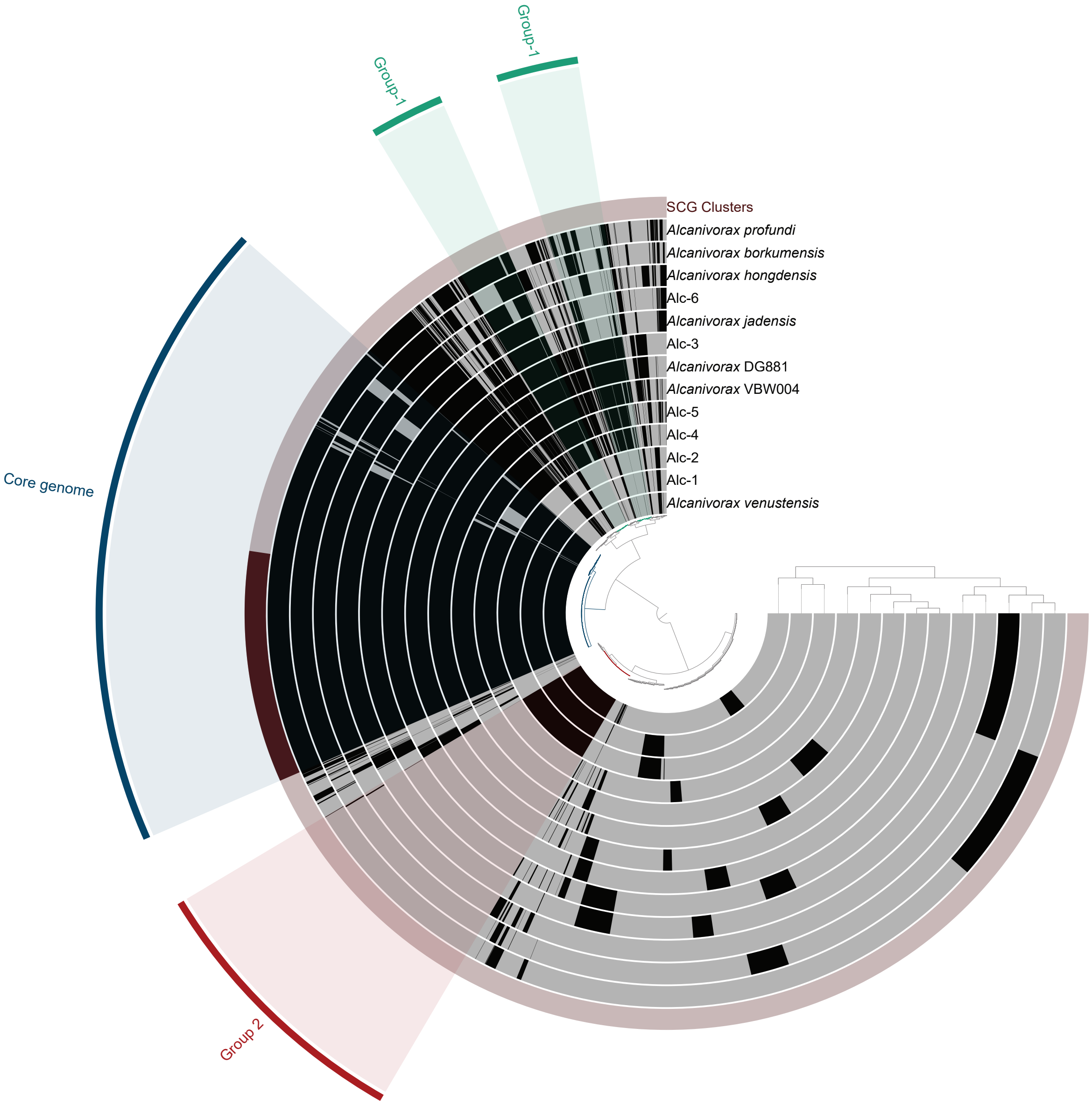

Figure S13. The pangenome of the *Alcanivorax* genus using *Alcanivorax* MAGs retrieved in this study and genomes of the most studied cultivated species including, *Alcanivorax borkumensis*, *Alcanivorax hongdensis*, *Alcanivorax jadensis*, *Alcanivorax profundi*, *Alcanivorax* strain DG881, *Alcanivorax* strain VBW001 and *Alcanivorax venustensis*. Black lines depict the occurrence of gene clusters. The "Core genome" section corresponds to the genes shared between all genomes. Group 1 corresponds to genes shared between all genomes but *Alcanivorax venustensis* and the closely-related MAGs, Alc-2 and Alc-1. Genes corresponding only to these three MAGs but not to other genomes, were grouped in Group 2. SCG clusters refer to single-copy gene clusters. The right-hand side section provides additional information on hierarchical clustering of the MAGs based on single-copy genes.

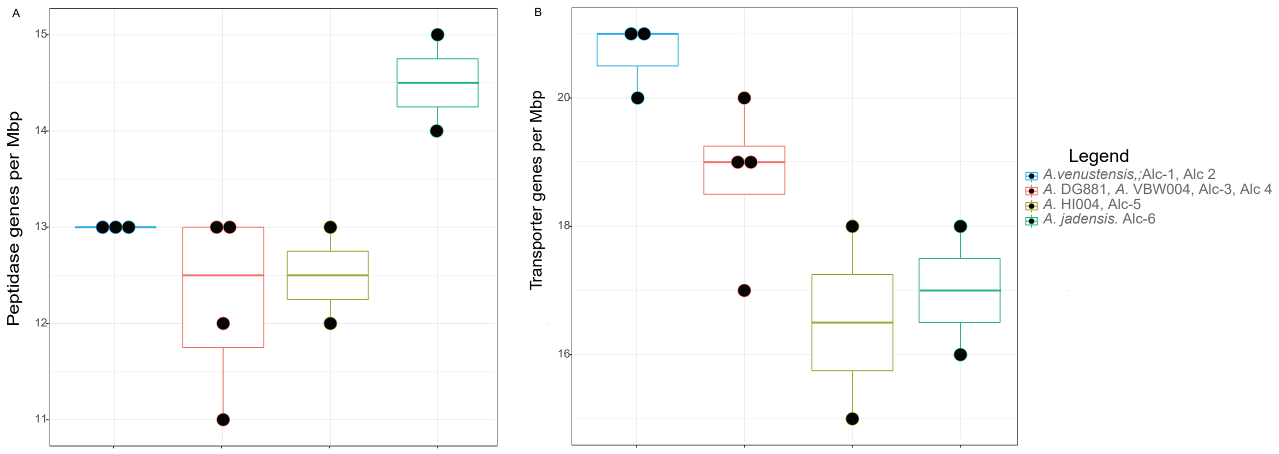

Figure S14. Comparison of peptidases and transporters gene per Mbp in *Alcanivorax* genomes.

Peptidases and transporters were annotated as described in Priest et al. (24).

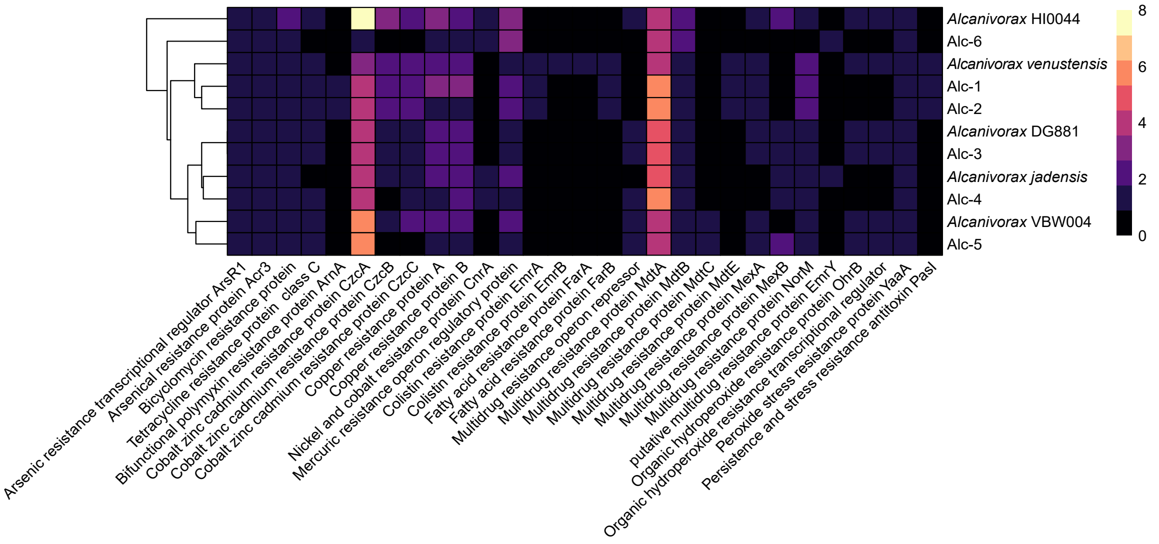

Figure S15. Heatmap of the resistance genes in *Alcanivorax* MAGs retrieved in this study and their closely-related cultivated species including *Alcanivorax* HI0044, *Alcanivorax venustensis*, *Alcanivorax* DG881, *Alcanivorax jadensis* and *Alcanivorax* VBW004. The legend represents the number of resistance genes in each MAG.

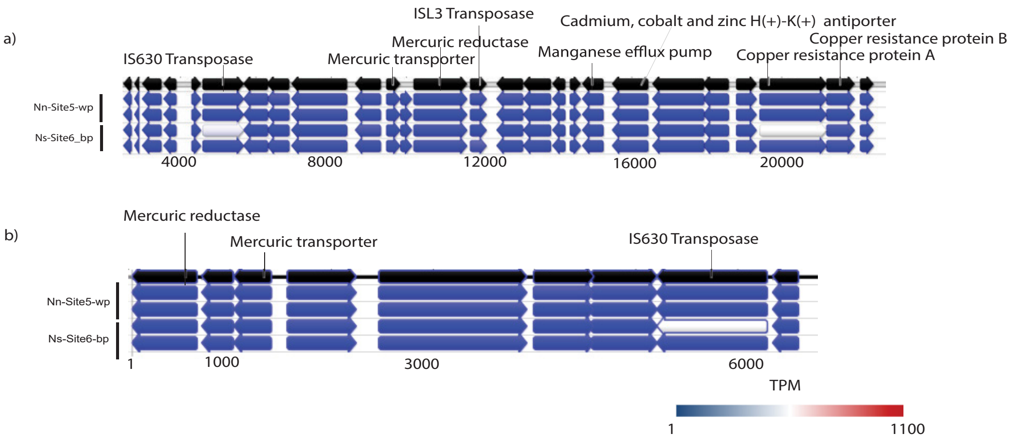

Figure S16. Mercuric reductase gene placement between transposases in a) Alc-1 and b) Alc-2. The genes are denoted by black arrows and the two replicates of each metatranscriptome (Nn-Site5-wp and Nn-Site6-bp) were mapped to them (identity=97%). The expression is denoted in four lanes of arrows beneath genes and their color indicates the transcript per million (TPM).

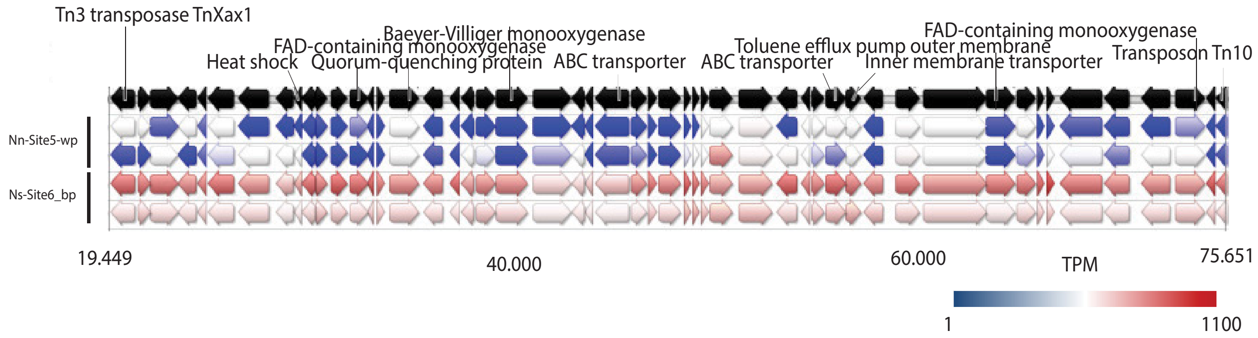

Figure S17. Placement of additional FAD-binding monooxygenase gene between transposases in Alc-3. The genes are denoted by black arrows. Metatranscriptomes of sample Nn-Site5-wp and Nn-Site6-bp (2x technical replicates per sample) were mapped to Alc-3 with a minimum identity of 97%. The expression is denoted in four lanes and their color indicates the transcript per million (TPM).

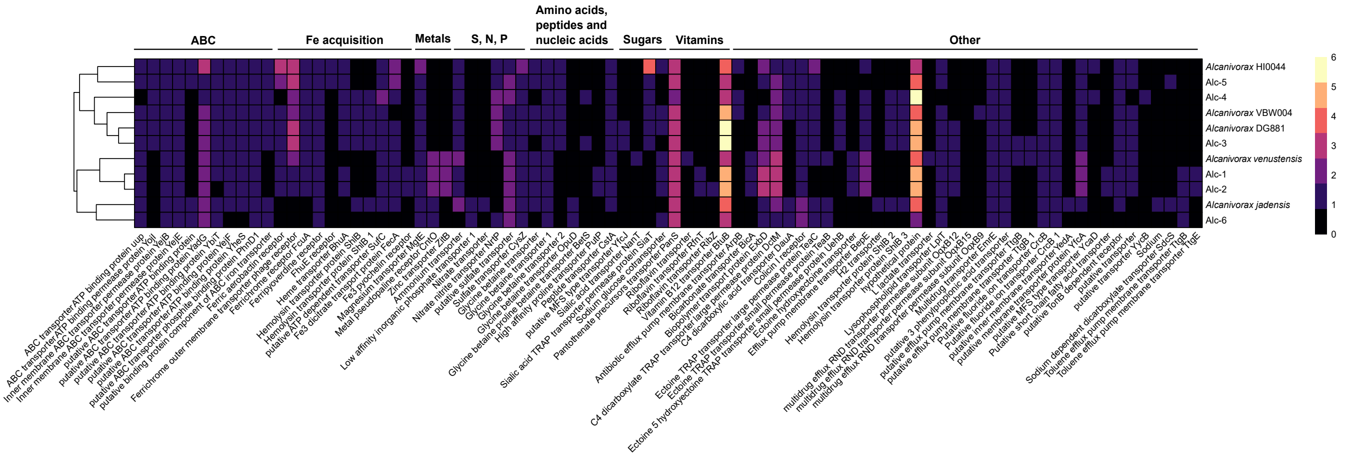

Figure S18. Heatmap of the transporters in *Alcanivorax* MAGs retrieved in this study and their closely-related cultivated species *Alcanivorax* HI004, *Alcanivorax venustensis*, *Alcanivorax* DG881, *Alcanivorax jadensis* and *Alcanivorax* VBW004. Legend represents the number of genes in each MAG.

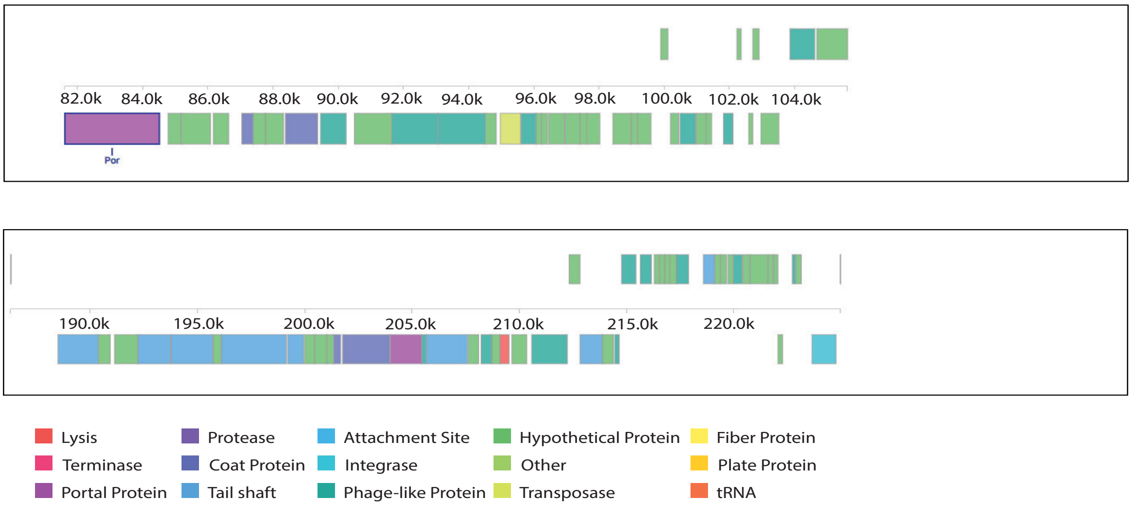

Figure S19. Viral sequences in Alc-3 analyzed using PHASTER (25). Both sequences had >90 score. Viral genes are depicted in different colors.

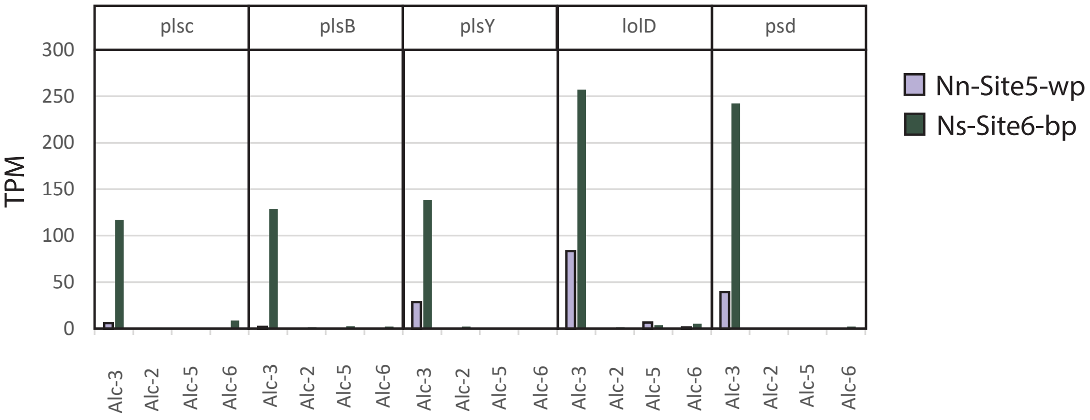

Figure S20. Expression of genes involved in biosurfactant synthesis. Genes analysed are *plsC* – Acetyltransferase domain protein, *plsB* – Glycerol-3-phosphate acyltransferase, *plsY* – Probable glycerol-3-phosphate acyltransferase, *lolD* – Lipoprotein.releasing system ATP-binding protein, *psd* – Phosphatidylserine decarboxylase. Transcripts were normalized to the length of the gene and total number of reads in metatranscriptoms (TPM).

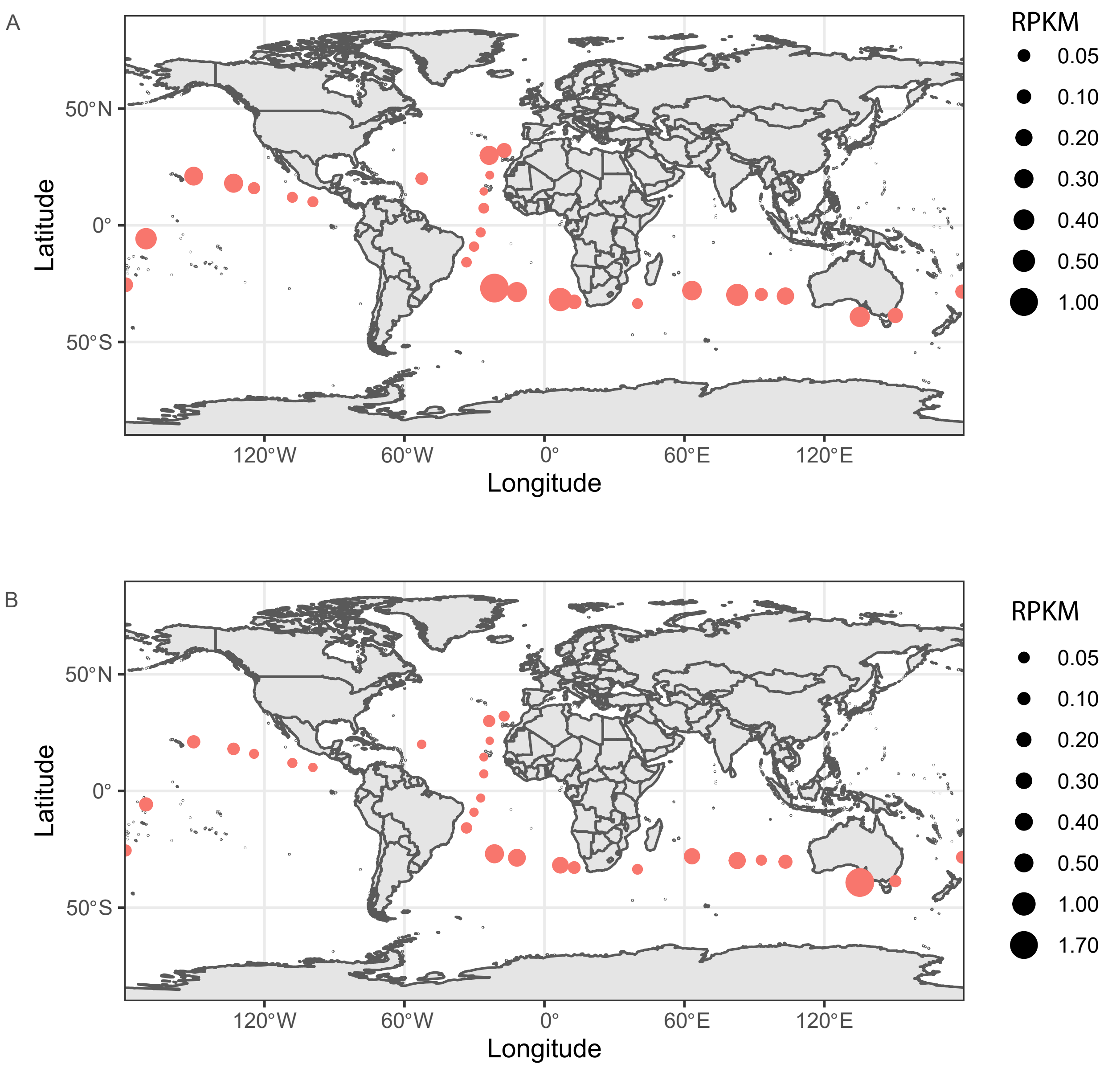

Figure S21. Abundance of A) Alc-3 and B) Alc-6 in Malaspina dataset (10). Reads were mapped unambiguously using BBmap (18) with 99% minimum identity. Abundance was calculated as RPKM.

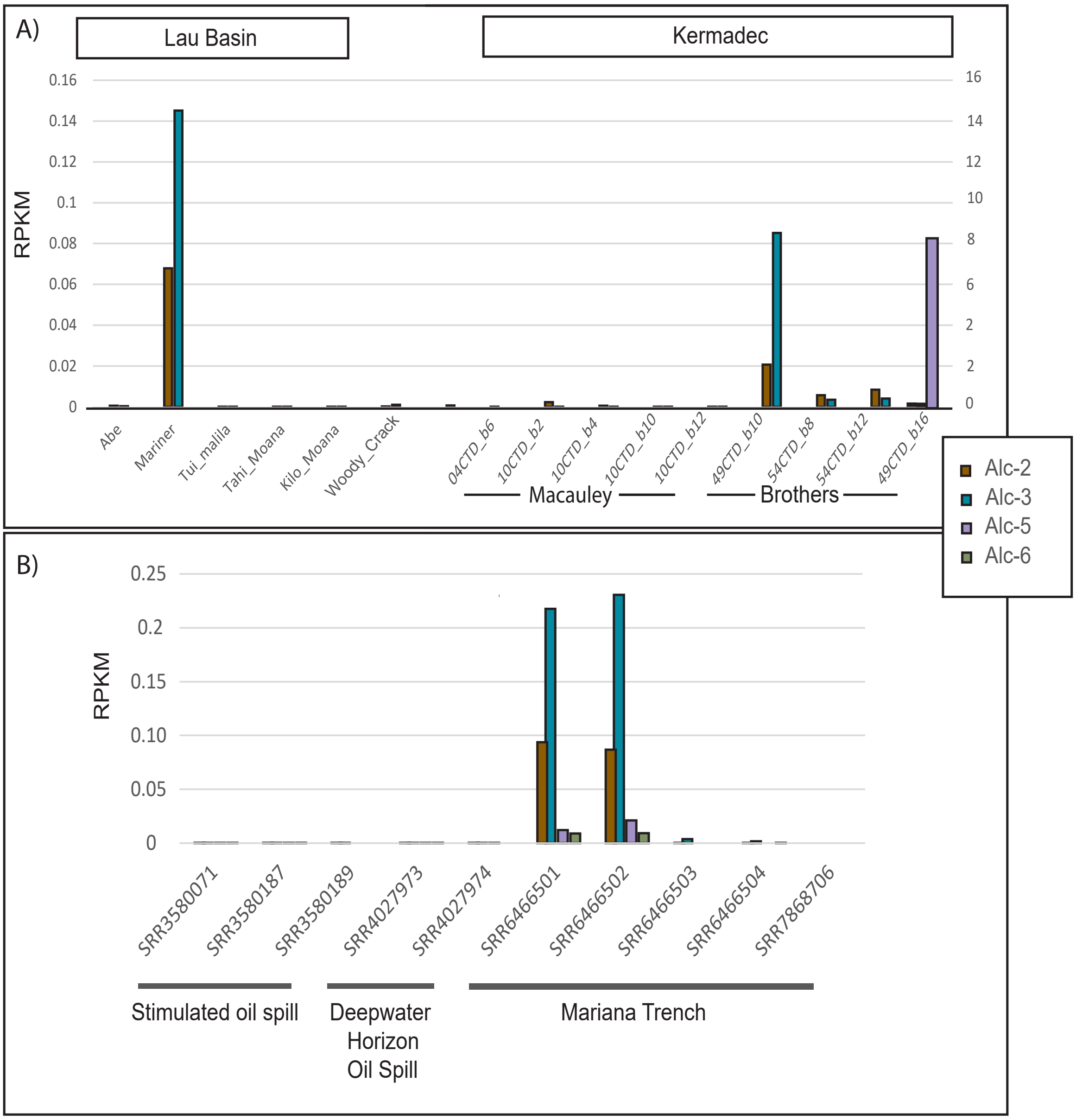

Figure S22. Abundance of *Alcanivorax* MAGs in other plumes and oil spill metagenomes. A) RPKM of MAGs in plumes of the Lau Basin as well as Brothers and Macauley volcano in the Kermadec arc. Sample 49CTD_b16 is represented on the secondary axes. B) RPKM of MAGs in stimulated and real oil spill systems and alkane-rich samples of Mariana Trench.

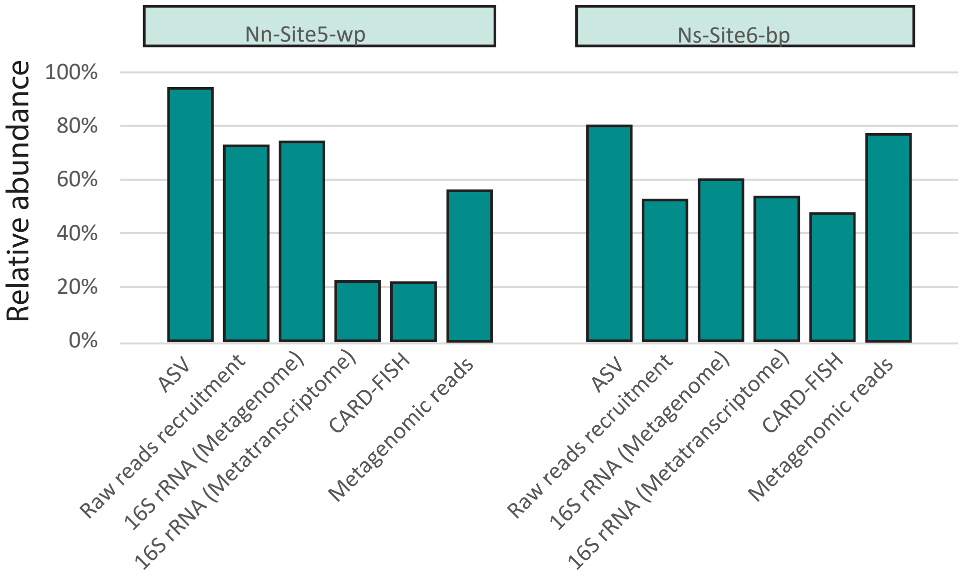

Figure S23. Comparison of *Alcanivorax* relative abundance between different cultivation-independent techniques. Raw read recruitment represent the fraction of metagenomics raw reads recruited on the *Alcanivorax* MAGs. 16S RNA was sorted from metagenomes and metatranscriptomes using SortMeRNA (19). Cells were visualized and counted using CARD-FISH with a mixture of ALV735 and ALV735-b probes. All metagenomics reads were taxonomically affiliated using Kaiju (26).
